# The Potential for Sample Testing at the Pen Level to Inform Prudent Antimicrobial Selection for Bovine Respiratory Disease Treatment: Investigations Using a Feedlot Simulation Tool

**DOI:** 10.3390/antibiotics14101009

**Published:** 2025-10-11

**Authors:** Dana E. Ramsay, Wade McDonald, Sheryl P. Gow, Lianne McLeod, Simon J. G. Otto, Nathaniel D. Osgood, Cheryl L. Waldner

**Affiliations:** 1 Western College of Veterinary Medicine, University of Saskatchewan, Saskatoon, SK S7N 5B4, Canada; 2 Department of Computer Science, University of Saskatchewan, Saskatoon, SK S7N 5C9, Canada; 3 Canadian Integrated Program for Antimicrobial Resistance Surveillance, Public Health Agency of Canada, Saskatoon, SK S7N 5B4, Canada; 4 HEAT-AMR (Human-Environment-Animal Transdisciplinary AMR) Research Group, School of Public Health, University of Alberta, Edmonton, AB T6G 2J7, Canada

**Keywords:** agent-based model (ABM), simulation model, antimicrobial resistance (AMR), antimicrobial use (AMU), bovine respiratory disease (BRD), diagnostic testing, feedlot cattle, veterinary medicine

## Abstract

**Background:** Antimicrobial drugs are used to treat bacterial diseases in livestock production systems, including bovine respiratory disease (BRD) in feedlot cattle. It is recommended that therapeutic antimicrobial use (AMU) in food animals be informed by diagnostic tests to limit the emergence of antimicrobial resistance (AMR) and preserve the effectiveness of available drugs. Recent evidence demonstrates preliminary support for the pen as a prospective target for AMR testing-based interventions in higher-risk cattle. **Methods:** A previously reported agent-based model (ABM) was modified and then used in this study to investigate the potential for different pen-level sampling and laboratory testing-informed BRD treatment strategies to favorably impact selected antimicrobial stewardship and management outcomes in the western Canadian context. The incorporation of sample testing to guide treatment choice was hypothesized to reduce BRD relapses, subsequent AMU treatments and resultant AMR in sentinel pathogen *Mannheimia haemolytica*. The ABM was extended to include a discrete event simulation (DES) workflow that models the testing process, including the time at sample collection (0 or 13 days on feed) and the type of AMR diagnostic test (antimicrobial susceptibility testing or long-read metagenomic sequencing). Candidate testing scenarios were simulated for both a test-only control and testing-informed treatment (TI) setting (n = 52 total experiments). Key model outputs were generated for both the *pen* and *feedlot* levels and extracted to data repositories. **Results:** There was no effect of the TI strategy on the stewardship or economic outcomes of interest under baseline ecological and treatment conditions. Changes in the type and number of uses by antimicrobial class were observed when baseline AMR in *M. haemolytica* was assumed to be higher at feedlot arrival, but there was no corresponding impact on subsequent resistance or morbidity measures. The impacts of sample timing and diagnostic test accuracy on AMR test positivity and other outputs were subsequently explored with a theoretical “extreme” BRD treatment protocol that maximized selection pressure for AMR. **Conclusions:** The successful implementation of a pen-level sampling and diagnostic strategy would be critically dependent on many interrelated factors, including the BRD treatment protocol, the prevalences of resistance to the treatment classes, the accuracy of available AMR diagnostic tests, and the selected “treatment change” thresholds. This study demonstrates how the hybrid ABM-DES model can be used for future experimentation with interventions proposed to limit AMR risk in the context of BRD management.

## 1. Introduction

The World Health Organization (WHO) describes antimicrobial resistance (AMR) as “one of the top global public health and development threats” [1]. Bacterial AMR was estimated to have contributed to nearly 5 million deaths in 2019 in a recent analysis of its global burden [2], a problem driven by misuse and overuse of antimicrobials in humans, animals, and plants [1,3]. Antimicrobial drugs are administered to food animals to prevent, control, and treat bacterial diseases, and their use is an integral part of industrialized livestock production systems [3,4]. Most antimicrobials sold worldwide are used in animals raised for food [5], making this sector a target for policies that limit inappropriate antimicrobial use (AMU) and the risks posed by AMR emergence [6,7]. In addition to the direct impacts of AMR on human and animal health, both Canadian [8] and international [9] action plans on AMR highlight how treatment failures linked to decreasing antimicrobial effectiveness drive production losses and food insecurity.

Bovine respiratory disease (BRD) continues to be the most common and costly disease of feedlot cattle in North America [10,11] and is the primary reason for the administration of individually dosed AMU in Canadian feedlots [12]. The likelihood of parenteral (i.e., injectable) AMU is influenced by BRD risk category, an assessment that includes factors related to animal age and weight, origin, clinical appearance, and previous management history [13]. In their study of AMU in 36 western Canadian feedlots, Brault et al. [14] reported that 95% of feedlot cattle categorized as “high risk” for respiratory disease received injectable antimicrobials to control or treat BRD. While the individual treatment of diseased cattle in beef production is both routine and required to support animal welfare, the emergence of resistant BRD pathogens can threaten the therapeutic efficacy of available antimicrobial drugs [15]. The direct and indirect impacts of BRD treatment failure were reviewed in [16] and include costs related to relapse treatments, the management of chronically ill animals, reduced salvage values, and increased mortality. Further, the repeated exposure of individual animals and their pen-mates to antimicrobials has the potential to exacerbate AMR [16].

Lhermie et al. [6] noted that antimicrobial therapy for bacterial infections is rarely preceded by confirmatory diagnostic and susceptibility testing in food animal production due to the limited availability, costs, and slow turnaround of such tests. Existing BRD treatment protocols in feedlot production rely on clinical signs and known risk factors rather than the laboratory testing of individual animals to inform AMU [17,18]. It has nevertheless been recommended that all antibiotic prescriptions in animals be informed by a diagnostic test, where available, to reduce unnecessary AMU and the risk of AMR [19]. In particular, the WHO guidelines advise that antimicrobials of critical importance to human medicine not be used in food-producing animals for disease control or treatment unless susceptibility testing indicates that it is the only option [20]. Given the complex pathogenesis and multifactorial etiology of BRD [21], timely and accurate therapy is considered necessary for a successful treatment outcome (i.e., a direct return to health [16]); however, there are currently no sufficiently sensitive, commercially available testing options that produce results with which to inform antimicrobial drug selection without a significant time delay [17,18,22].

Modern feedlot operations manage cattle as groups in pens; calves in shared pens are expected to be more similar in terms of disease and AMU exposures than those in different pens. While it is not currently practical to test individual calves before treatment in large commercial feedlots, a recent study [17] considered the potential to use laboratory data from their pen mates or contemporary management cohorts to inform BRD antimicrobial treatment protocols. Indeed, the perceived inability to extrapolate diagnostic test results to the entire pen or herd was reported as a barrier to testing uptake in [6]. Recent studies from our research group demonstrate broad support for the pen-cohort as a prospective target for testing-based interventions [23,24]. For example, Abi Younes and colleagues report on the significant associations between pen-level culture and susceptibility results from previous testing and the corresponding findings in BRD-affected calves from the same pen in [24]. As with any effort to support the judicious use of antimicrobials in livestock production, consideration should be given to the practical integration and potential impacts of candidate pen-level sampling and testing strategies to inform antimicrobial drug selection.

In [25], Gröhn discusses how the systems science approach can be used to optimize intervention strategies in food animal systems. Specifically, he advocates the idea that “integrating modeling and mathematics with biological studies” is the best way to address the challenges of maintaining a safe food supply [25]. In contrast to conventional, single-discipline methods, dynamic simulation modeling methods incorporate the complexities of biological systems and can anticipate the upstream and downstream consequences of changes to those systems [25,26]. Our research group developed a stochastic, agent-based model (ABM) to examine the dynamics of population-level AMR in a sentinel BRD pathogen in pens of higher-risk cattle on a typical western Canadian feedlot [27]. The ABM is unique in its hierarchical depiction of behavioral units at multiple levels (i.e., pathogen within animal, pen and feedlot “agents”) [28], and in its use of diverse data sets to explain emergent system phenomena (e.g., AMR prevalence). The foundational study in [27] demonstrated the model’s value as a tool for exploring questions related to antimicrobial stewardship in the context of BRD management.

The Pan-Canadian Action Plan on Antimicrobial Resistance suggests that evidence-based guidance on antimicrobial therapy and the appropriate use of diagnostics should be tailored to local AMR risks and burdens where possible [8]. The feedlot simulation tool [27] was used in this work to investigate the potential for various pen-level sampling and laboratory testing-informed BRD treatment strategies to favorably impact selected stewardship and management outcomes in the western Canadian context. The ABM was extended in this study to include an “agent” that facilitates the sampling and testing process; the agent comprises a discrete event simulation (DES) workflow, a dynamic modeling method that is useful for following individual entities (i.e., test samples) through event-driven processes [26]. The updated tool is thus a hybrid ABM-DES model, drawing on the strengths of each approach to advance our understanding of how testing-informed treatment could support prudent AMU in feedlots.

## 2. Materials and Methods

### 2.1. Model Description

A complete model description is available in Supplementary File S1 and follows the Overview, Design concepts, and Details (ODD) protocol for detailing ABMs [29,30]. It has been updated to reflect the additions to this model since it was first published in January 2025 [27]. As with the initial model calibrations and experiments described in that work [27], the experiments reported here were performed with exclusively higher-risk steers entering a small- to mid-sized feedlot in western Canada.

### 2.2. Purpose

A previously described [27] stochastic, continuous-time, hybrid ABM was modified and then used to examine the effect of laboratory testing-informed selection of antimicrobial treatments for BRD at the *pen* level on select BRD, AMU, and AMR outputs of interest. BRD relapses associated with AMR-linked therapeutic failure (i.e., the failure of a BRD case to respond to a particular therapy due to resistance in the causative organism) were posited to increase the number of antimicrobial uses. AMR prevalence was likewise theorized to increase in response to selective pressure. Conversely, the incorporation of sample testing to guide antimicrobial treatment choice was hypothesized to reduce BRD relapses, subsequent AMU treatments, and resultant AMR. This work will compare the impact of pen sampling strategies and AMR diagnostic tools on key antimicrobial stewardship metrics and feedlot economic outcomes.

### 2.3. Key Assumptions

The simplifying assumptions which informed the construction and scope of this model have been explored in detail [27]. The following section will highlight where additional assumptions were made to facilitate experimentation with various sampling and testing strategies to inform treatment selection.

#### 2.3.1. Model Configuration and Diagnostic Paradigms

A previous study determined that the model variants which included the impact of contagious acquisition on population-level AMR within the feedlot offered a stronger fit to empirical data than those that relied only on selection associated with AMU [27]. Therefore, a configuration which allowed for both AMU-linked selection and transmission of AMR (referred to in this work as the “both” configuration) was assumed to be the most appropriate for experimentation with the feedlot simulation tool in this work.

There were two AMR diagnostic paradigms explored in this study. The “phenotypic” approach concerns the susceptibility of the sentinel pathogen to a particular antimicrobial class as determined by culture-dependent antimicrobial susceptibility testing (AST) [24]. The “genotypic” approach concerns the presence or absence of a particular antimicrobial resistance gene (ARG) in the sentinel pathogen as determined by long-read metagenomic sequencing (MS) [31]. The model was calibrated to the time-varying proportion of resistant *Mannheimia haemolytica* isolates across the feeding period in [27]; given the lack of published data, a similar longitudinal data set could not be generated for the proportion of *M. haemolytica* isolates with known ARGs. The calibrated AMR selection, waning, and transmission parameters from [27] were therefore assumed to be reasonable proxies for the acquisition and loss of genes that confer resistance to the associated antimicrobial class in this work. It follows that for the purposes of this study, we made the conservative assumption that the pathogen’s resistance genotype and phenotype were perfectly concordant. In other words, the presence of a known ARG always confers clinical resistance to the associated antimicrobial class.

The selection of *M. haemolytica* as the sentinel pathogen in this work reflects the availability and reliability of temporal resistance prevalence data from feedlot cattle for this organism [32,33,34,35,36,37,38]. As in our previous work, the phenotypic and genotypic resistance status of *M. haemolytica* was broadly assumed to be representative of the most clinically relevant AMR in the nasopharyngeal microbiome, with the exception of *Mycoplasmopsis bovis* [21,35,36].

#### 2.3.2. Responsiveness of BRD Incidence to AMR

The model’s AMR responsiveness mechanism allows for complex feedback between AMU and AMR in sentinel pathogen *M. haemolytica* over the feeding period. More specifically, the model assumes that (1) calves arrive at the feedlot with AMR typical of that previously reported in similar populations [32,33,34,35,36,37,38]; (2) selection arising from AMU in combination with transmission increases detectable AMR (phenotype) and ARGs (genotype) in *M. haemolytica*; and (3) the presence of detectable AMR or ARGs in *M. haemolytica* decreases the success of antimicrobial treatments for BRD. The success of BRD metaphylaxis is responsive to resistance at the *calf* level. Calves with on-arrival resistance to the antimicrobial drug used for metaphylaxis experience first cases of BRD at the same rate as those who receive “no metaphylaxis” [27]. Conversely, the success of BRD therapy is responsive to resistance at the *pen* level at the time of treatment. Antimicrobial treatments delivered to calves who develop BRD will fail (i.e., animals will relapse) at a probability equivalent to the pen-level prevalence of AMR, if the pen-level prevalence of resistance to the administered drug exceeds the historical rate of retreatment. The functionality of the mechanism was previously demonstrated in a thought experiment [27] that simulated the conditions required for maximum AMR responsiveness (i.e., high levels of resistance to the therapeutic options). The logic underscoring these assumptions forms a critical component of the investigations in this work.

#### 2.3.3. Sampling and Testing in Advance of the Need to Treat

The foundation for the strategy to inform treatment extends the AMR responsiveness mechanism to include the potential for laboratory testing. It assumes that the pen-level prevalence of resistance to the first-line antimicrobial drug in the treatment protocol can be determined in advance of the need to treat for BRD. This strategy could theoretically limit the risk arising from AMR-linked treatment failure at the time of treatment) [17,23]. When the pen-level prevalence of resistance to the default drug meets the prescribed threshold at the time of sample (see Section 2.3.5), the calves in that pen that develop a first or subsequent case of BRD will be directed to receive a pre-specified alternative option at the time of treatment.

The experiments reported here involve one of two sampling time points selected for distinct reasons, including (1) the ease and feasibility of sampling (i.e., at the time of animal processing on feedlot arrival, referred to in this work as 0 days on feed (DOF)), and (2) the likelihood of resistance at sampling (i.e., at 13 DOF, after the highest impact of tulathromycin metaphylaxis on the respiratory bacteria [39] but before the peak of first BRD cases expected for fall-placed calves [27,40]). Notably, the time at sampling can be varied as desired in future experiments with the model. In the absence of resistance data to inform treatment (i.e., before the test is performed and/or before the results are available from the diagnostic laboratory), the alternative options are not used by the model and treatment for BRD will continue to fail at the probability described in the Section 2.3.2.

#### 2.3.4. Exposure to Antimicrobials

The AMU options for prophylaxis, metaphylaxis, and therapy in the model were first reported in [27] and are fully detailed in Supplementary File S1. The available selections are common in western Canadian feedlot medicine and were developed in consultation with feedlot experts.

**Figure 1:**
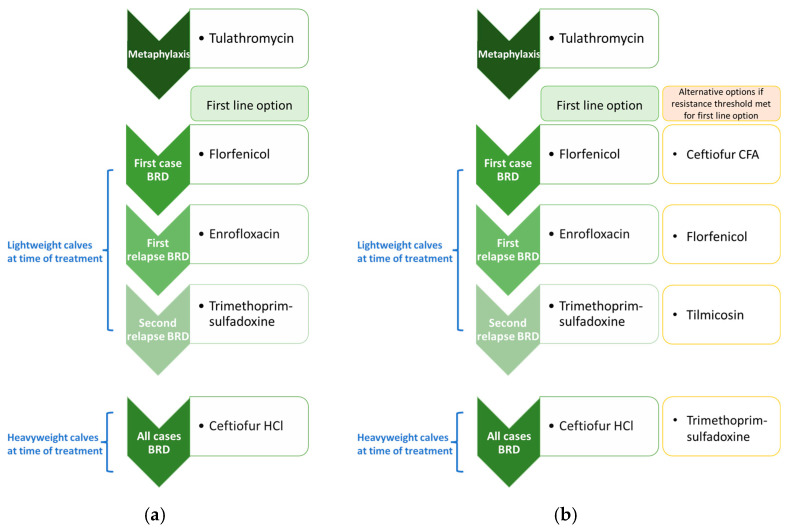
Metaphylaxis and BRD treatment protocol in the baseline scenarios for high-risk feedlot calves in the (**a**) test-only experiments. The first-line BRD control and treatment protocol for the baseline scenarios was developed in consultation with feedlot experts [27]. Where there were multiple treatment options for an indication in [27] (e.g., first BRD relapses), we selected only one option that remained constant across experiments for the purposes of this study (the parameter permitting variation in AMU protocols across unique runs in a single experiment was disabled). This ensured that relative changes in the median values for outputs of interest could be attributed to the experimental conditions rather than stochastic variation across realizations and (**b**) testing-informed treatment experiments. The alternative BRD treatment protocol in the testing-informed treatment experiments included licensed antimicrobials purposively selected to better distinguish between the impacts of the intervention on successive BRD therapies (i.e., changes in the number of antimicrobial uses by class) in the simulated data. The alternative drugs were not necessarily selected for the purpose of good antimicrobial stewardship.

For the purposes of this study, the AMU protocol for each indication was probabilistically selected and then held constant across experiments (i.e., the parameter permitting variation in AMU protocols across unique runs in a single experiment was disabled). This choice ensured that relative changes in the median values for outputs of interest (e.g., number of antimicrobial uses by class) could be attributed to the experimental conditions rather than stochastic variation across realizations. The metaphylaxis and first line options for first and subsequent cases of BRD in the baseline treatment protocol scenarios are reported in Figure 1a (test-only experiments); the alternative options reported in Figure 1b (testing-informed treatment experiments) were licensed antimicrobials purposively selected to better distinguish between the impacts of the intervention on successive BRD treatments in the simulated data. Practically, this improved our ability to verify that the diagnostic testing-informed treatment mechanism was working as intended.

**Figure 2:**
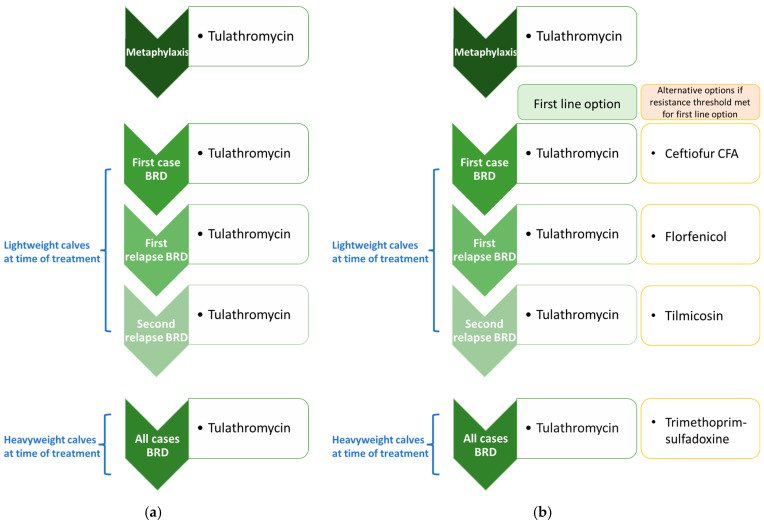
Metaphylaxis and BRD treatment protocol in the theoretical “extreme macrolide use” scenarios for high-risk feedlot calves in the (**a**) test-only experiments. The first-line BRD control and treatment protocol for the “extreme macrolide use” scenarios was designed to maximize selection pressure for AMR and mirrors the conditions for the thought experiment investigated in [27], and (**b**) testing-informed treatment experiments. For ease of comparison, the alternative BRD treatment protocol in the testing-informed treatment experiments for the “extreme macrolide use” scenarios were the same as in the baseline scenarios (Figure 1b).

In a different subset of experiments, the baseline BRD treatment protocol was replaced with a theoretical “extreme 15-membered ring macrolide use” protocol to create scenarios with very high selection pressure for AMR (see Section 2.8). Tulathromycin was used for metaphylaxis and first-line therapy for all BRD cases in the extreme protocol (Figure 2a); for ease of comparison, the alternative options (Figure 2b) remained the same as in the baseline protocol.

The fixed treatment protocols used in these experiments for non-BRD prophylactic and therapeutic indications are reported in Table 1.

#### 2.3.5. Treatment Change Threshold

The threshold at which the tested pen-level prevalence of resistance to the first-line drug triggered a change to that pen’s treatment protocol was set to 25% (i.e., 5 positive tests of 20 tested animals) in most of the scenarios examined here. In the initial model, the “historical rate of retreatment (i.e., probability of first BRD relapse)” was the threshold above which BRD treatments could fail due to pen-level AMR; the value (21.6%) was specific to high-risk calves with first cases of BRD after tulathromycin metaphylaxis and derived from empirical data supplied by a partner veterinary practice [27]. The “treatment change” threshold used in these experiments was intentionally selected to marginally exceed that probability of all-cause treatment failure in the baseline model [42]. An alternative to this assumption was explored in sensitivity analyses in the present study, and the ability to modify the relevant threshold is equally available for future experimentation.

### 2.4. Testing Agent

**Figure 3:**
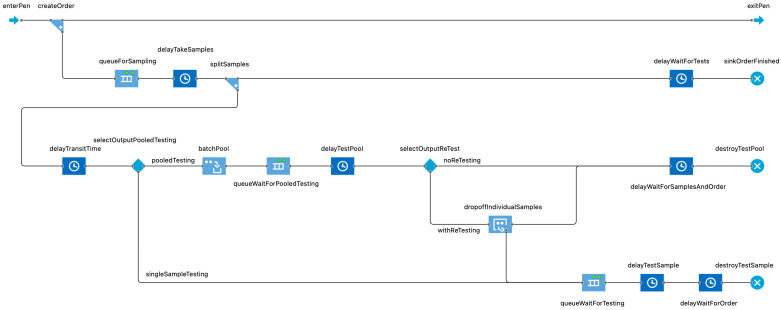
Discrete event simulation (DES) component of the hybrid agent-based model, detailing the process of sampling and diagnostic AMR testing of samples from calves in home pens. A *Pen* agent enters the system and has a “test order” created for it; the default setting dictates that 20 randomly selected animals in the “home pen” be sampled and tested to determine the pen’s AMR status. The workflow follows the samples through the event-driven testing process, which incorporates modifiable time delays for collection, transit and processing. The time required to ship and process the nasopharyngeal samples delays the availability of test results with which to make informed treatment decisions.

A *Testing* agent has been added to the model’s infrastructure to facilitate the comparison of different pen sampling strategies and AMR diagnostic tools. The agent contains a DES workflow that models the sampling and testing process (Figure 3). As part of this process, a *Pen* agent figuratively enters the system and has a “test order” created for it; the order dictates that 20 randomly selected animals in the pen be sampled (at either 0 or 13 DOF) and tested (by either AST or MS) to determine the pen’s AMR status. The number of sampled animals per pen can be varied as desired in future experiments with the model and is explored in sensitivity analyses in the present study. The DES workflow likewise allows the user to incorporate and vary time delays for sample collection, sample transit, and sample processing as required. It is assumed that the time required to ship and process the nasopharyngeal samples delays the availability of test results with which to make informed treatment decisions.

The *Testing* agent also contains information on diagnostic test performance (i.e., test sensitivity and test specificity). In the baseline scenarios in this work, it was assumed that the theoretical AMR test had perfect (100%) sensitivity and specificity. In subsequent experiments, these measures of test performance were replaced with empirical values for AST and MS derived from Bayesian latent class analyses of field data [43] (see Section 2.5). The samples in these simulation experiments were processed and tested individually, but the option exists in the model to pool samples prior to diagnostic testing if desired. Individual tests can be grouped (“batched”) according to pool size, which is specified by a modifiable parameter if pooled testing is enabled.

### 2.5. Input Data

Most of the model’s inputs were extensively reported and discussed in [27], consistent with best practices that emphasize the importance of transparency in model parameterization [44,45,46]. All parameter values for the present model are reported in the ODD protocol in Supplementary File S1. New or updated model inputs for this series of experiments are displayed in Table 2. In particular, the updated average daily gain (ADG) parameters for both healthy and diseased cattle (see *Cattle agent parameters* in Table 2) were informed by proprietary data from private, western Canadian feedlot operations representing over 200,000 animals. For cattle with first or subsequent cases of BRD or arthritis, the impact of disease on growth rate is expressed as an absolute decrease in ADG relative to healthy animals. Reductions in ADG were treated as additive for calves affected by both diseases over the feeding period.

The probabilities of phenotypic resistance to each antimicrobial class in *M. haemolytica* isolates at feedlot arrival are summarized in Table 3. In the experiments with baseline on-arrival resistance, the probabilities were equal to the values used in the calibration experiments in [27]. The probabilities of incoming resistance were increased in a subset of “worst-case” scenario experiments designed to simulate higher levels of AMR to the first-line therapeutic options (see Section 2.8). In the “high on-arrival resistance” experiments, the values were set to the upper confidence intervals of the population-averaged prevalence estimates for *M. haemolytica* isolates (n = 70) from calves sampled in 2022 as part of the Canadian Feedlot Antimicrobial Use and Antimicrobial Resistance Surveillance Program (CFAASP) [47].

#### 2.5.1. Testing Agent Parameters

The time delay parameters governing the DES workflow in the newly implemented *Testing* agent are reported in Table 2 (see Section 2.5.1). The delays were informed by expert opinion deriving from empirical observations of the sample collection, transport and processing steps in a multi-year research study [31,32] and CFAASP [37,48] projects. The time required for each of these steps was equal for the diagnostic test types examined in this series of experiments (AST and MS); in future experiments, these parameters could be modified to reflect local circumstances (e.g., laboratory availability) and advances in sequencing methods that reduce the time to results [49]. For the purpose of these experiments, it was assumed that diagnostic test results were available on-line to inform treatment decisions immediately following the sample processing step (i.e., there was no delay for the reporting or communication of test results).

#### 2.5.2. Diagnostic Test Characteristics

The diagnostic test parameters (i.e., empirical estimates for test sensitivity and specificity) for both AST and MS are reported in Table 4. Within the phenotypic paradigm, the test parameters define the ability of AST to detect phenotypic resistance to targeted antimicrobial classes (macrolides, tetracyclines) in *M. haemolytica* isolates from nasopharyngeal samples (Table 4). Within the genotypic paradigm, the test parameters define the ability of MS to detect the presence of representative ARGs (*msrE-mphE*, *estT*, *tetH*) in *M. haemolytica* reads from nasopharyngeal samples.

The low, median, and high estimates for sensitivity and specificity in Table 4 were obtained from latent class analyses (LCA) of field data from over 900 feedlot calves sampled at least three times as part of a multi-year research project [43]. Specifically, the estimates were derived from two-test, three- or five-population Bayesian latent class models (BLCMs) in the absence of a “gold standard” [50] for detecting resistant *M. haemolytica* from deep nasopharyngeal swabs of feedlot cattle.

The reference genes selected for LCA (Table 4) were those detected most frequently by long-read MS in [31,51] or identified in the retrospective analysis in [52] and known to confer resistance to the reference drugs representing antimicrobial classes of interest in the model [27]. Where the BLCM reached a stable solution for the combination of reference drug/gene, the estimates for test sensitivity and specificity are reported as Bayesian medians with 95% credible intervals (CrI). The “low” and “high” estimates for each characteristic are equal to the lower and upper limits of the CrI, respectively. For classes of antimicrobials with very low levels of phenotypic and/or genetic resistance (e.g., fluoroquinolones and cephalosporins), the BLCMs failed to provide reliable estimates of test sensitivity and specificity. The low, median, and high values for these parameters were therefore conservatively set to equal those of the reference drug (for AST) or gene (for MS) with the *lowest* BLCM-derived estimate.

**Table 4:** Diagnostic test sensitivity and specificity values used in the experiments for the detection of phenotypic resistance to select antimicrobial drugs (AST) and the detection of select antimicrobial resistance genes in *Mannheimia haemolytica* from nasopharyngeal samples (Metagenomics).

			Diagnostic Test Characteristics					
			Sensitivity Estimate (%)			Specificity Estimate (%)		
Antimicrobial Class	Diagnostic Test Type	Reference Drug ^1^ or Gene ^2^	Low	Median	High	Low	Median	High
*Classes with BLCM-derived estimates ^3^*								
**15-membered ring macrolides**	AST	Tulathromycin	73%	80%	86%	99%	100%	100%
	Metagenomics	*msrE-mphE*	56%	62%	69%	96%	98%	99%
**16-membered ring macrolides**	AST	Tilmicosin	10%	23%	38%	99%	100%	100%
	Metagenomics	*estT*	22%	43%	65%	98%	99%	100%
**Sulfonamides**	AST	Sulfadimethoxine	83%	90%	98%	98%	99%	100%
	Metagenomics	*sul2*	57%	63%	69%	90%	92%	95%
**Tetracyclines**	AST	Oxytetracycline	11%	18%	25%	99%	100%	100%
	Metagenomics	*tetH*	66%	82%	95%	94%	97%	99%
*Classes without BLCM-derived estimates ^4^*								
**Diaminopyrimidines**	AST	Trimethoprim	10%	18%	25%	98%	99%	100%
	Metagenomics	*dfrA14*	22%	43%	65%	90%	92%	95%
**Cephalosporins**	AST	Ceftiofur	10%	18%	25%	98%	99%	100%
	Metagenomics	*blaROB-2*	22%	43%	65%	90%	92%	95%
**Fluoroquinolones**	AST	Enrofloxacin	10%	18%	25%	98%	99%	100%
	Metagenomics	*gyrA mutation*	22%	43%	65%	90%	92%	95%
**Phenicols**	AST	Florfenicol	10%	18%	25%	98%	99%	100%
	Metagenomics	*floR*	22%	43%	65%	90%	92%	95%

^1^ The antimicrobial drug with the most complete data set (i.e., one or more raw data points for each time point) was selected to represent all others in its class in the calibration experiments in [27]. ^2^ The antimicrobial resistance gene detected most frequently by metagenomic sequencing in [31,51,52] and known to confer resistance to the reference drug was selected for Bayesian latent class analysis in [43] and/or these Monte Carlo experiments. ^3^ Bayesian latent-class model (BLCM)-derived estimates for sensitivity and specificity were available for select reference drugs and resistance genes only [43]. The low and high estimates for each test characteristic are equal to the lower and upper limits of the 95% credible interval for the estimate, respectively. ^4^ Bayesian latent-class model (BLCM)-derived estimates for sensitivity and specificity were not available for select reference drugs and resistance genes with low prevalences of phenotypic and/or genotypic resistance in [43]; for these classes, the low, median, and high values for sensitivity and specificity were conservatively set to equal that of the reference drug (for AST) or gene (for metagenomic sequencing) with the **lowest** BLCM-derived estimate.

### 2.6. Key Model Outputs

There were several emergent model outputs prioritized for summary and comparison across the scenarios examined in this work. From an AMR risk-based perspective, key outputs included the simulated prevalences of resistance to select classes of antimicrobials at (1) 50 DOF (the data-informed time point at which most of the high-risk animals who will get sick have been treated for a first case of BRD [27,40,53]); (2) 70 DOF (the intermediate-term data-informed time point with the strongest empirical data in [27]); and (3) 170 DOF (the data-informed time point closest to the end of the feeding period and animal slaughter). A related output concerned the percentage of n = 5000 realizations where the simulated prevalence exceeds some antimicrobial class-specific benchmark value (Table 5), purposively selected for distinct time points to highlight meaningful changes in the distribution of AMR prevalence across different testing scenarios. For 50 and 70 DOF, the benchmark values were set to the population-averaged resistance prevalence estimates for *M. haemolytica* isolates from calves sampled at rehandling in 2023 as part of CFAASP [12,54]. For 170 DOF, the benchmark values reflected the relative importance of the antimicrobial class to human medicine and the risk posed by resistance in the late feeding period [55]; the selected values are considered “rare”, “very low” or “low” per the levels of resistance defined by the European Food Safety Authority [56].

Priority disease and treatment-related outputs of interest included (1) the number of BRD relapses (i.e., second, third, or fourth cases of BRD) by the end of the simulated feeding period, and (2) the cumulative number of drug uses by antimicrobial class and category at 170 DOF. The model’s testing-informed treatment logic works to limit the risk of AMR-linked treatment failure, and these outcomes may therefore be impacted by the introduction of sample testing to guide treatment choice for first and subsequent BRD cases under specific conditions. Collectively, these outputs generate important insights related to both antimicrobial stewardship practices and economic outcomes on western Canadian feedlots. Additional outcomes with economic implications that could be affected by BRD treatment-focused interventions and which were selected for summary included the number and destination of finished calves, the number of days to finishing weight, and the number of deaths by cause at the end of the feeding cycle.

### 2.7. Model Verification

Owing to the recent availability of AMR surveillance data from CFAASP [12,47,54], we conducted a preliminary external validation step to confirm that the model configuration selected for these experiments (i.e., the “both” configuration which allowed for both AMU-linked selection and transmission of AMR) could reproduce outputs comparable to real-world observations. The population-averaged resistance prevalence estimates (and 95% CIs) for *M. haemolytica* isolates from calves sampled at rehandling in 2022 (median DOF = 14), 2023 (median DOF = 70) and 2024 (median DOF = 45) were graphed against the Monte Carlo outputs simulated for selected antimicrobial classes in [27] (see Supplementary File S2: Figures S2.1–S2.5). Importantly, these data were not used to calibrate the model, and offered the opportunity to evaluate the model’s performance against an independent data set. For most classes and time points, the 95% CIs for the CFAASP estimates overlapped in whole or in part with the 95% prediction intervals for the “both” configurations in [27], strengthening our confidence in the model’s reliability as an experimental tool. The model performed poorly for 16-membered ring macrolides at 14 DOF; this was not unexpected given our low confidence in the raw data for this time point [27].

A full description of the summary outputs generated for multirun configurations is available in [27]. In Supplementary File S2: Table S2.1, we demonstrate that the model outputs are consistent with the empirically derived model inputs for a test-only control simulation. As in our previous work, these outputs were critical for checking and troubleshooting the model’s logic with the addition of the *Testing* agent and testing-informed treatment infrastructure. Novel outputs related to the “testing” component were added to the summary outputs and included the count of single tests performed, the count of tests that were positive for resistance by antimicrobial class (both *pen* and *feedlot* levels), and the count of pens where the number of positive tests met the “treatment change” threshold (n ≥ 5). The test positivity percentage by antimicrobial class was calculated in a subsequent step for both the *pen* and *feedlot* levels; this summary statistic was particularly useful in verifying that the timing and function of the cattle testing mechanism was working as intended.

**Figure 4:**
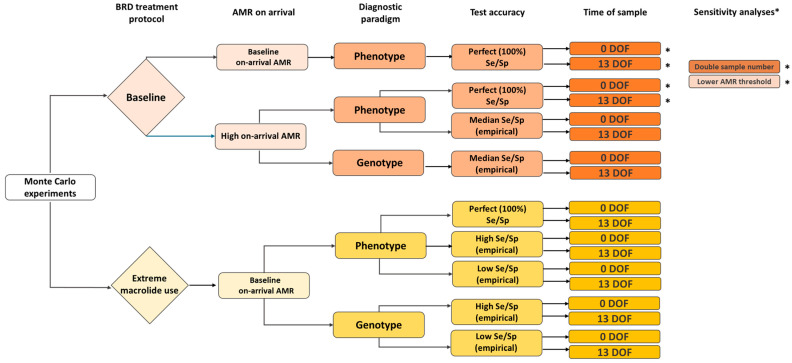
Summary of cattle sampling and AMR testing scenarios analyzed in this series of Monte Carlo experiments; each scenario is simulated for both a test-only (i.e., no treatment changes based on test results) and a testing-informed treatment setting (n = 52 total experiments). Scenarios were purposively selected to highlight changes to key outputs, and feature variations in BRD treatment protocol (baseline vs. extreme), on-arrival AMR prevalence (baseline vs. high), diagnostic test type and accuracy, and time of test (feedlot arrival vs. 13 DOF). Sensitivity analyses were performed for scenarios which used the baseline BRD treatment protocol and a perfect AMR test (marked with *).

For each cattle sampling and AMR testing scenario of interest (Figure 4), a “test-only” control was performed to better isolate the impact of testing conditions (e.g., AMR prevalence, test accuracy) from those of “testing-informed” changes to the treatment protocol. It likewise ensured that simulated testing errors were not impacting the downstream outcomes of primary concern (i.e., antimicrobial uses, BRD relapses). The test positivity rates for the experiments and their “test-only” controls were compared for equivalence to highlight how the test conditions in different combinations did or did not trigger the decision rule (i.e., “switch to alternative if resistance to first-line option meets the treatment change threshold”). The treatment and morbidity outcomes for the experiment-control pair could theoretically differ *if* the “treatment change” threshold (25%) was met.

### 2.8. Summary of Monte Carlo Experiments

A summary of the sampling and testing scenarios examined with the model is provided in Figure 4. The collection of experiments was refined over several iterative steps, such that the final selection better highlights the potential of testing-informed treatment or meaningful changes in the key outputs where they exist. Specifically, many of the experiments were designed to simulate the conditions for maximum responsiveness to AMR in one of two ways: (1) the on-arrival prevalences of resistance to the therapeutic options were increased to empirical “worst-case” levels for a subset of experiments using the baseline (calibration) treatment protocol for BRD (see Section 2.5 and Table 3), or (2) the baseline BRD treatment protocol was replaced with a theoretical “extreme 15-membered ring macrolide use” protocol that maximized selection pressure for AMR when on-arrival resistance was held at baseline (see Section 2.3.4 and Figure 2). Such high responsiveness scenarios allowed us to better examine the impact of imperfect diagnostic tests on the expected outcomes, given that the changes to key outputs were otherwise negligible under baseline conditions.

The chart in Figure 4 highlights the variations across scenarios in terms of treatment protocol (baseline vs. extreme), on-arrival AMR prevalence (baseline vs. high), diagnostic paradigm (phenotype vs. genotype), diagnostic test accuracy (perfect vs. empirical) and time of test (feedlot arrival vs. 13 DOF). Note that when test accuracy was perfect, the choice of diagnostic paradigm did not functionally change the model; perfect tests were thus only completed for phenotype and not genotype scenarios. Each scenario was simulated for both a test-only (i.e., no treatment changes based on test results) and a testing-informed treatment setting (n = 52 total experiments, including sensitivity analyses). Experiments were run for n = 5000 realizations over a one-year model time horizon. Simulated outputs were generated for both the *pen* and *feedlot* levels and summarized in an MS Excel workbook with the medians, interquartile range (IQR), and 95% prediction intervals (2.5th and 97.5th percentiles).

### 2.9. Analysis of Model Output

To facilitate the comparison of results across scenarios, key model outputs were extracted from the summary reports to data repositories in MS Excel (see Supplementary Files S3 and S4). These outputs provide a framework for making relative comparisons across sampling and testing scenarios and are not intended to make exact predictions concerning the AMR risk or economic outcomes of interest. Where meaningful differences in the outputs of interest owing to either (1) testing variations or (2) hierarchical unit (*pen* vs. *feedlot*) were detected, these results were visualized with figures created in R (version 4.3.2) or MS Excel.

#### Sensitivity Analyses

Additional scenarios were examined as part of a sensitivity analysis to evaluate the impact of changes to key testing parameters on the outputs for select experiments that used the baseline BRD treatment protocol (see final column in Figure 4). To assess the impact of sample number on the diagnostic test positivity rate, the first such experiment doubled (from 20 to 40) the number of sampled cattle per pen at both testing times (0 and 13 DOF). In a second such experiment, we assessed the impact of lowering the threshold of pen-level resistance that triggered a treatment change (from 25% to 10%, or 2 of 20 sampled calves) at both time points. The sensitivity experiments were run for n = 5000 realizations over 1 year, and the outputs (medians, IQRs and 95% prediction intervals) were likewise extracted to the data repository in MS Excel for comparison (see Supplementary Files S3 and S4).

## 3. Results

Output data for the “baseline” and “extreme” BRD treatment protocol scenarios were stored as tables in separate repositories and can be viewed in Supplementary Files S3 and S4. Meaningful changes across experiments in the outputs of interest will be highlighted in this section, as will key takeaways related to the impact of sampling and testing conditions.

### 3.1. Scenarios Using the Baseline AMU Protocol

**Figure 5:**
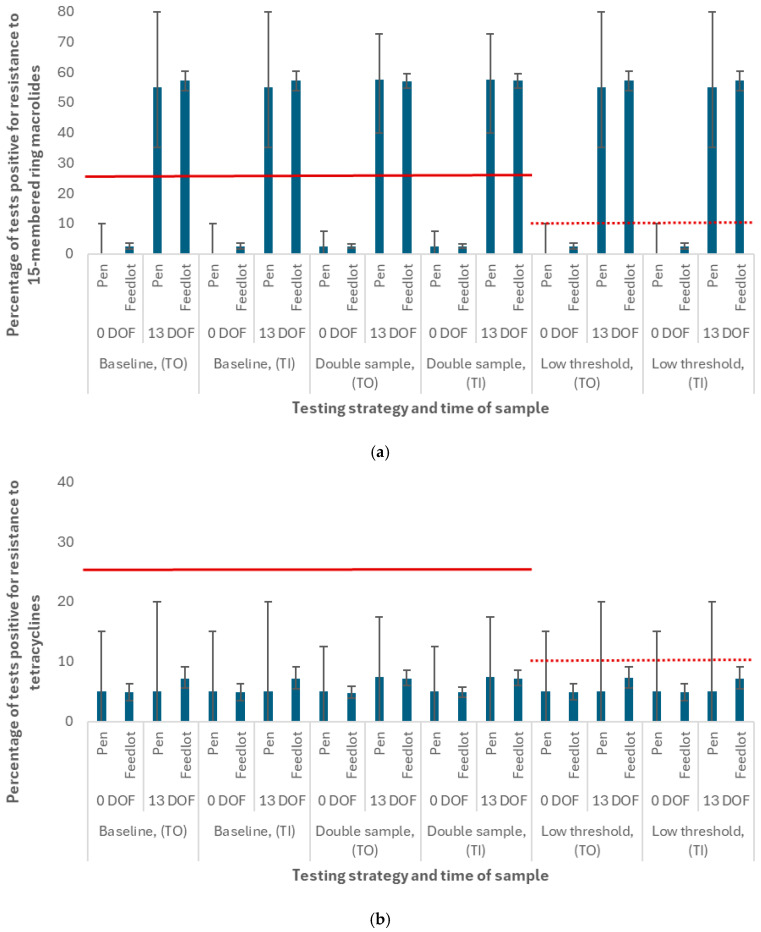
Percentage of tests that were positive for phenotypic resistance to (**a**) 15-membered ring macrolides, top and (**b**) tetracyclines, bottom, for different testing strategies using the “baseline” BRD treatment protocol (see Figure 1) and a “perfect” test. The results for the sensitivity analyses are reported in the figure as “double sample” (where the number of sampled cattle is doubled from 20 to 40) and “low threshold” (where the “treatment change” threshold is lowered from 25% to 10%). For each scenario, the median percentage and 95% prediction interval across n = 5000 realizations results are reported at both the *pen* and *feedlot* levels for the test-only control experiments (TO) and the testing-informed treatment experiments (TI). The solid red line represents the default 25% “treatment change” threshold; the dashed red line represents the 10% “treatment change” threshold in the sensitivity analysis.

The diagnostic test positivity percentages for the baseline scenario were reported for selected antimicrobial classes at both the *pen* and *feedlot* levels in Figure 5. This figure likewise illustrated (1) the impact of doubling the number of sampled cattle per pen on the median test positivity percentage and 95% prediction interval, and (2) the impact of lowering the “treatment change” threshold on the frequency of switching to the alternative treatment option. Importantly, the AMR test in these baseline scenarios had perfect sensitivity and specificity, and the on-arrival resistances matched those in the calibration setting.

As expected, the percentage of tests that were positive for phenotypic resistance to 15-membered ring macrolides (Figure 5a) and tetracyclines (Figure 5b) were identical for the testing-informed treatment (TI) experiments and their test-only (TO) controls in each scenario. This observation was an important first step in verifying that the testing mechanism was working as intended. The median AMR test positivity percentage was significantly different for 15-membered ring macrolides when the test was performed at 0 DOF (2.5% at the *feedlot* level for all scenarios) than when it was performed at 13 DOF (57.1%), reflecting the sharp, near-term increase in resistance to this class following metaphylactic exposure to tulathromycin at feedlot arrival. The difference in median AMR test positivity percentage for tetracyclines was comparatively small, increasing from 4.9% (at the *feedlot* level for all scenarios) when the test was performed at 0 DOF to just 7.2% when the test was performed at 13 DOF. This difference reflects the more gradual increase in and later peak prevalence (50 DOF) of resistance to tetracyclines as compared to macrolides.

The impressive differences in the variation in this outcome (i.e., the range of AMR test positivity percentages across 5000 realizations of the model) by the unit of analysis (*pen* vs. *feedlot*) is an important takeaway from Figure 5. For example, while the median test positivity percentage for 15-membered ring macrolides at 13 DOF was similar for the *pen* (55.0%) and *feedlot* (57.1%) levels in the baseline scenario, the 95% prediction interval was substantially wider at the *pen* level (35.0–80.0% vs. 53.9–60.3%). A similar but less sizeable difference in test positivity variation was observed for tetracyclines when the test was performed at 13 DOF (0–20.0% vs. 5.6–9.1% for the *pen* and *feedlot* levels, respectively). For all antimicrobial classes except fluoroquinolones (data not visualized), the difference in output variation for pens vs. feedlots was greater when the test was performed at 13 DOF than when it was performed at 0 DOF.

Under baseline (calibration) conditions, there were nil or negligible impacts of the testing-informed treatment strategy on the antimicrobial stewardship or economic outcomes of interest. In particular, the simulated prevalences of resistance (50 and 170 DOF), the number of antimicrobial uses by class, the number of BRD relapses and deaths, and the days to finishing weight were unaffected by the availability of diagnostic results, regardless of the time of test. The pen-level prevalence of resistance to the default drug classes used to treat first, second, and third cases of BRD (i.e., phenicols, fluoroquinolones, and potentiated sulfonamides, respectively) never reached the “treatment change” threshold (25%) in this scenario, as evident in the low median test positivity percentage (<5%) for these antimicrobials (data available in Supplementary File S3).

#### 3.1.1. Sensitivity of Outputs to Testing Parameters in Baseline Scenario

For most combinations of antimicrobial class and testing time point, doubling the per-pen sample number had the anticipated impact of improving the precision of the test positivity percentage estimate (i.e., the 95% prediction intervals were narrower, see “double sample” experiments in Figure 5). The change in estimate precision was greater at the *pen* than the *feedlot* level, given that the benefit of additional samples diminishes when the sample size is already large (n = 960 samples from 48 pens in the *feedlot* at baseline). The median test positivity percentage at the *pen* level was increased by 2.5% for select classes when the sample was doubled, such that the diagnostic result more accurately reflected the simulated prevalence of resistance at 0 DOF (15-membered ring macrolides only) or 13 DOF (15- and 16-membered ring macrolides and tetracyclines) reported in [27]. Increasing the sample size had no effect on the treatment and morbidity outcomes of interest in the baseline scenarios (i.e., outputs downstream of the testing process were robust to changes in this assumption).

Similarly, lowering the “treatment change” threshold from 25% to 10% had no effect on the priority outcomes for the TI experiments under baseline conditions. In some minority proportion of pens, the tested resistance to 15-membered ring macrolides (0 DOF) and tetracyclines (0 or 13 DOF) newly exceeded the revised threshold where it previously did not. This is evident where the upper limits of the 95% prediction intervals meet or span the red line in the “low threshold” experiments (Figure 5). There were no pens where the tested resistance to phenicols, fluoroquinolones, and potentiated sulfonamides newly exceeded the revised threshold (i.e., the entire distributions of test positivity values for these first-line treatment classes are less than 10%, data available in Supplementary File S3). Consequently, there was no increase in alternative treatment use relative to baseline.

#### 3.1.2. Impact of Strategy When Incoming Resistance Is High

**Figure 6:**
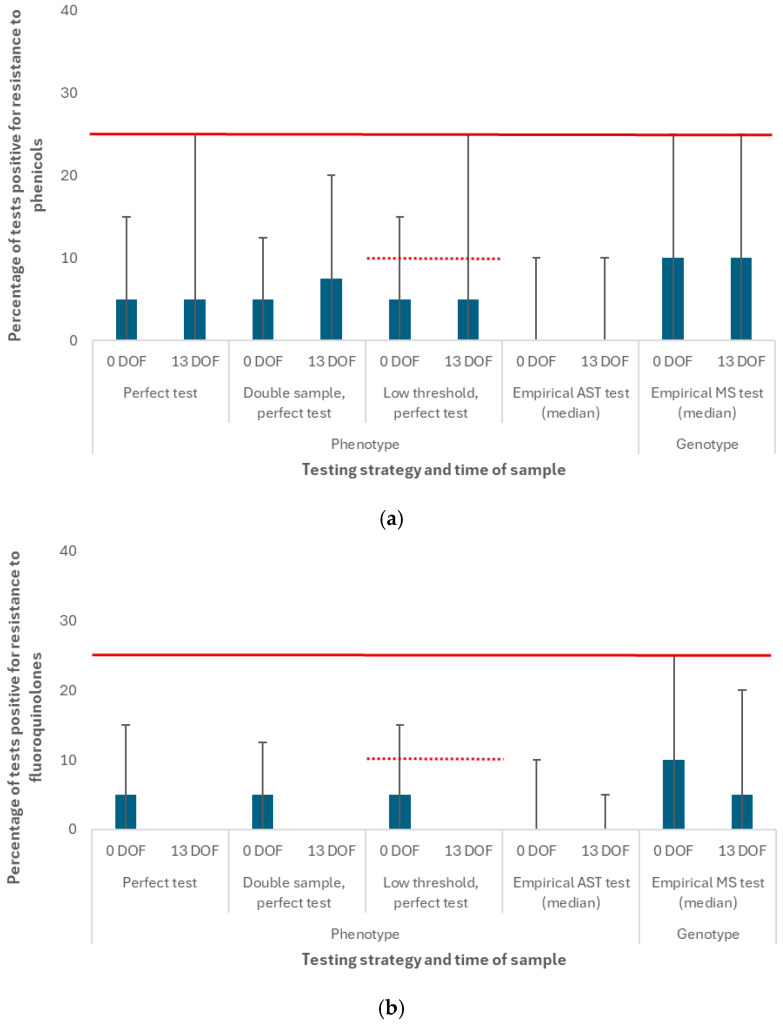
Percentage of tests that were positive for resistance to (**a**) phenicols, top; (**b**) fluoroquinolones, bottom, for different testing strategies using the “baseline” BRD treatment protocol (Figure 1) with *high on-arrival resistance* (Table 3). The results for the sensitivity analyses are reported in the figure as “double sample” (where the number of sampled cattle is doubled from 20 to 40) and “low threshold” (where the “treatment change” threshold is lowered from 25% to 10%). For each scenario, the median percentage and 95% prediction interval across n = 5000 realizations results are reported at the *pen* level only for the testing-informed treatment experiments. The solid red line represents the default 25% “treatment change” threshold; the dashed red line represents the 10% “treatment change” threshold in the sensitivity analysis. Empirical sensitivity and specificity values for the antimicrobial susceptibility (AST) and metagenomic sequencing (MS) tests are in Table 4.

The diagnostic test positivity percentages for the scenarios with high on-arrival resistance are reported for select antimicrobial classes at the *pen* level only in Figure 6. The percentage of tests that were positive for phenotypic or genotypic resistance to phenicols (default drug class for first BRD cases, Figure 6a), fluoroquinolones (default drug class for second BRD cases, Figure 6b), and potentiated sulfonamides (default drug class for third BRD cases, see Supplemental File S3) were identical for the TI experiments and their TO controls in each scenario. The TO data was omitted from this figure to simplify the visualization.

The median AMR test positivity percentage for phenicols at the *pen* level was the same when a perfect test was performed at 0 or 13 DOF (5%), but the upper limit of the 95% prediction interval was different (15% vs. 25%, respectively); the “treatment change” threshold was only met at 13 DOF for a small proportion (2.5%) of total pens when a perfect test was used (Figure 6a). The empirical AST test for phenicols has low estimated sensitivity (18%) but high specificity (99%) at the sample level, and the test failed to detect phenotypic resistance to florfenicol under the testing conditions (i.e., 20 samples per pen with increased background resistance) in more than 50% of the realizations. The median AMR test positivity percentage was 0% (0%, 10%) at the *pen* level at both 0 and 13 DOF, and the “treatment change” threshold was not met. The empirical MS test for phenicols has comparatively higher sensitivity (43%) but lower specificity (92%), and therefore the test also detected genotypic resistance to phenicols (i.e., the *floR* gene) where it was not expressed; the median AMR test positivity percentage was 10% (0%, 25%) at the *pen* level at both 0 and 13 DOF, and the “treatment change” threshold was met at both times for a small proportion (2.5%) of total pens.

The median AMR test positivity percentage for fluoroquinolones at the *pen* level was higher at 0 DOF (5%) than 13 DOF (0%) when a perfect test was used, though the “treatment change” threshold was not met at either time point (Figure 6b). The estimated sensitivity and specificity values for the empirical AST and MS tests for fluoroquinolones were equal to those for phenicols (Table 4); as with phenicols, the empirical AST test failed to detect phenotypic resistance to enrofloxacin at 0 DOF (0%) in more than 50% of the realizations. Likewise, the empirical MS test also detected genotypic resistance to fluoroquinolones (i.e., a *gyrA* gene mutation) where it was not expressed; the median AMR test positivity percentage at the *pen* level was 10% (0%, 25%) at 0 DOF and 5% (0%, 20%) at 13 DOF. The “treatment change” threshold was thus newly exceeded at 0 DOF for a small proportion of pens (2.5%) when the imperfect MS test was used. The 95% prediction intervals for tested fluoroquinolone resistance were wider at 0 DOF than 13 DOF (i.e., there was more variation in the outcome at the earlier time point) across all perfect and empirical test types.

**Figure 7:**
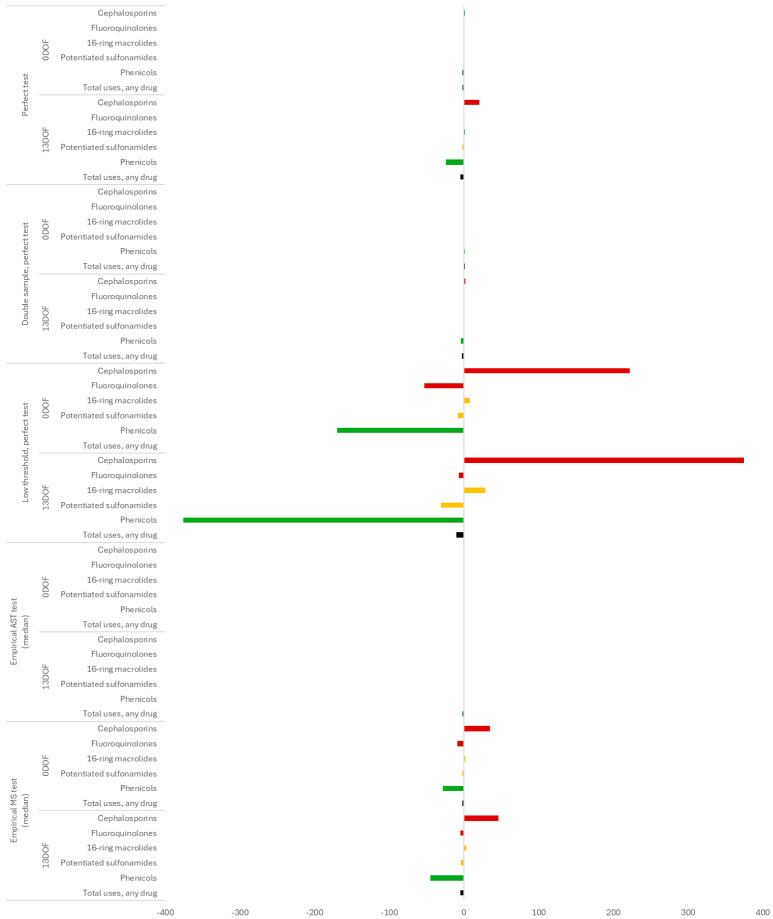
Change in the median number of drug uses ^1^ for treating BRD by antimicrobial class ^2,3^ across n = 5000 realizations for intervention experiments relative to the “test-only” (i.e., no testing-informed treatment) control. Results are reported for different testing-informed treatment strategies using the “baseline” BRD treatment protocol (Figure 1) with *high on-arrival resistance* (Table 3). The color of the bar corresponds to the category of importance to human medicine (Table 5), where the red, yellow and green bars represent classes belonging to Categories I, II and III, respectively ^4^. ^1^ There were 9600 fall-placed steer calves placed in 48 “home pens” in the simulation feedlot (i.e., 200 calves per pen). When the on-arrival resistance was high, there were 1108 first cases of BRD in the “test-only” control (11.5% of animals entering the feedlot). By 170 DOF, there were 1088, 242, 86, and 11 uses of phenicols, fluoroquinolones, trimethoprim–sulfadoxine and cephalosporins in the “test only” experiment, respectively, for all indications detailed in [27]. ^2^ Changes in the median number of 15-membered ring macrolide uses were excluded from this figure. This drug class (i.e., tulathromycin) was administered as metaphylaxis in every experiment in this study and was therefore not used as a first-line or alternative treatment for BRD. The median number of 15-membered ring macrolide uses could not be impacted by the testing-informed treatment strategies investigated in this work. ^3^ We expected to see decreases in the median number of uses of first-line BRD classes (e.g., phenicols, fluoroquinolones and potentiated sulfonamides for first, second and third cases in lightweight cattle) and corresponding increases in the median number of uses of alternative BRD classes (e.g., cephalosporins, phenicols and 16-membered ring macrolides for first, second and third cases in lightweight cattle) if the testing-informed treatment strategy was having an impact. Note that a net decrease for phenicols would be expected in this circumstance given that its increase in use for second treatments was small relative to its decrease in use for first treatments. ^4^ The alternative drugs were purposively selected to better distinguish between the impacts of the intervention on successive BRD therapies (i.e., changes in the number of antimicrobial uses by class) in the simulated data. The alternative drugs were not necessarily selected for the purpose of good antimicrobial stewardship.

Where the 95% prediction intervals for tested phenicol, fluoroquinolone or potentiated sulfonamide resistance met or exceeded the “treatment change” threshold, we expected to see shifts in the numbers of antimicrobial uses by class for the TI experiments (i.e., the test information would trigger a change in the treatment protocol). The change in median number of antimicrobial uses by class for the TI scenarios with high on-arrival resistance were reported at the *feedlot* level in Figure 7. The outputs were compared to a TO control where the median number of florfenicol, enrofloxacin, and trimethoprim–sulfadoxine uses for the 9600 calves placed in the feedlot were 1089, 242, and 87, respectively, through 170 DOF. There were no changes in use when a perfect test was performed at 0 DOF; when the perfect test was performed at 13 DOF, there were 25, 1 and 3 fewer median uses of florfenicol, enrofloxacin and trimethoprim–sulfadoxine, respectively. When the imperfect and less specific MS test was used, we observed additional decreases in the number of uses of these drugs at both testing time points (0 and 13 DOF) relative to the perfect test scenarios. Decreases in the use of first-line BRD treatment drugs were mostly offset by increases in the number of median uses of the designated alternatives in this experiment, ceftiofur (cephalosporins) and tilmicosin (16-membered ring macrolides); the net decreases in total uses of any drug were ≤5 when the MS test was used. No changes in use were observed for either testing time point when the AST test was used.

There were nil or negligible impacts of the testing-informed treatment strategy on the simulated prevalences of resistance (50 or 170 DOF) or the number of BRD relapses and deaths, even where the diagnostic results precipitated changes in the number and type of antimicrobial uses to treat BRD (data not visualized, see Supplementary File S3). Temporary increases in the percentage of *pens* and *feedlots* across n = 5000 realizations with detectable resistance (>0%) to cephalosporins (the alternative treatment class for first BRD relapses) were observed at 50 DOF when the perfect test was performed at 13 DOF, and when the empirical MS test was performed at 0 or 13 DOF. Importantly, the upper limits of the 95% prediction intervals for the median prevalence of resistance never exceeded 1%, and the effect had largely disappeared by 70 DOF. The median prevalences of resistance across the feeding period were unaffected for all other antimicrobial classes with changes in use. Further, the number of BRD relapses remained the same (n = 366) regardless of the time or type of diagnostic test performed.

There were substantial increases in the median *pen*-level prevalences of resistance at 50 DOF to select classes of antimicrobials when the incoming resistance was high (data not visualized, see Supplementary File S3). A comparison of the TO versions of these scenarios revealed particularly notable increases for phenicols (0% vs. 19.1%) and trimethoprim (0.5% vs. 24.6%), classes with low mean waning rates in the model (calibrated in [27]). Increases were also observed to a lesser extent for tetracyclines (18.6% vs. 24.6%). By 170 DOF, the impact of high incoming AMR on the pen-level prevalences of resistance to these classes was negligible (i.e., absolute differences ≤ 1%). There were approximately 70 more BRD relapses in the TO “high incoming AMR” scenario compared to its “baseline” counterpart (n = 296 relapses). This was entirely attributable to the increased likelihood of metaphylactic failure and first BRD cases when resistance to tulathromycin is high at arrival.

#### 3.1.3. Sensitivity of Outputs to Testing Parameters When Incoming Resistance Is High

As with the “baseline” scenarios displayed in Figure 5*,* doubling the per-pen sample number improved the precision of the test positivity percentage estimate (see “double sample” experiments in Figure 6) for most combinations of antimicrobial class and testing time point. For phenicols (Figure 6a) and potentiated sulfonamides (see Supplemental File S3), these improvements in precision meant that the upper limits of the 95% prediction intervals for this outcome no longer met or spanned the “treatment change” threshold at 13 DOF (i.e., relative to the standard 20 samples per pen). Consequently, shifts in the number of antimicrobial uses by class were no longer observed for the TI experiments when a perfect test was performed at 13 DOF (Figure 7), and the antimicrobial use profile reverted to that for the TO control setting.

Lowering the “treatment change” threshold from 25% to 10% had a substantial impact on the type and number of antimicrobial uses by class for the TI experiments when the incoming resistance was high (see “low threshold” experiments in Figure 7). When the test was performed at 0 DOF, the 95% prediction intervals for the tested resistance to phenicols and fluoroquinolones newly exceeded the revised threshold where they previously did not (Figure 6). In the TI scenario, there were 170 and 53 fewer median uses of florfenicol and enrofloxacin, respectively; decreases in the use of these classes were offset by increases in the median number of uses (n = 222) of ceftiofur. When the test was performed at 13 DOF, a greater proportion of pens (25% vs. 2.5%) met the threshold for treatment change for phenicols and potentiated sulfonamides (i.e., more of the distribution of test positivity values for these classes lie above the red line in Figure 6). Consequently, there were substantially more changes (n = 375) from florfenicol (default drug) to ceftiofur (alternative drug) for first cases of BRD in this TI scenario; there was likewise a small (n = 10) net decrease in total uses of any drug.

Despite these changes in the AMU profile, the AMR and morbidity outcomes of interest were only minimally affected by lowering the “treatment change” threshold (i.e., outputs downstream of the treatment process were robust to changes in this assumption, see Supplementary File S3). At both testing time points, there were temporary increases in the percentage of *pens* and *feedlots* across n = 5000 realizations with detectable resistance to cephalosporins (>0%) at 50 DOF for the TI scenarios; this increase was greater when the test was performed at 0 DOF than at 13 DOF (7.5% vs. 10% of *pens* with detectable cephalosporin resistance, respectively). The effect had largely disappeared by 70 DOF. There was no meaningful impact of this change on the number of BRD relapses or deaths, chronic pen usage, DOF to finishing weight, or number of finished calves.

### 3.2. Scenarios Using the Extreme Macrolide Use Protocol

**Figure 8:**
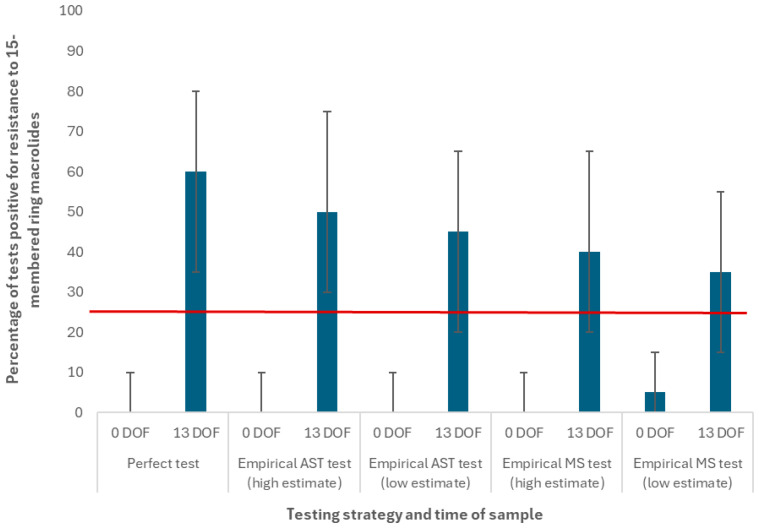
Percentage of tests that were positive for resistance to 15-membered ring macrolides for different testing strategies using the “extreme macrolide use” BRD treatment protocol (Figure 2). For each scenario, the median percentage and 95% prediction interval across n = 5000 realizations results are reported at the *pen* level only for the testing-informed treatment experiments. The solid red line represents the default 25% “treatment change” threshold. Empirical sensitivity and specificity values for the antimicrobial susceptibility (AST) and metagenomic sequencing (MS) tests are in Table 4.

The diagnostic test positivity percentages for different testing strategies using the “extreme macrolide use” treatment protocol are reported for 15-membered ring macrolides at the *pen* level only in Figure 8. At 0 DOF, the 95% prediction interval for tested 15-membered ring macrolide resistance did not meet or exceed the “treatment change” threshold for any combination of test and sensitivity/specificity estimate. When the “low” empirical estimates for sensitivity (56%) and specificity (96%) were used for the MS test at 0 DOF, the median test positivity percentage was higher (5%) than for the other variations (0%, see Figure 8). Compared to the results for the perfect test, this indicates that the sequencing test detected genotypic resistance to 15-membered ring macrolides (i.e., the *msrE-mphE* operon) where it was not expressed at 0 DOF. When test specificity is imperfect and the prevalence is low, a low positive predictive value is expected.

When the test was performed at 13 DOF, the median test positivity percentage exceeded the “treatment change” threshold for all combinations of test and sensitivity/specificity estimate. The test positivity medians and 95% prediction intervals were higher for the AST than for the MS test, given that even the “low” empirical estimate for AST sensitivity (73%) was higher than the “high” empirical estimate for MS (69%). Incremental reductions in the test positivity percentage are visualized in Figure 8 and associated with stepwise decreases in the empirical test sensitivity values for 15-membered ring macrolides summarized in Table 4. Based on these results, we expected to see corresponding decreases in the number of 15-membered ring macrolide uses for all variations in the tests performed at 13 DOF. The change in the median number of total drug uses for the TI scenarios (i.e., relative to a TO control scenario) are reported in Table 6.

Relative to the TO control, there were approximately 1050 (10%) fewer median uses of 15-membered ring macrolides by 50 DOF in the TI scenario where a perfect test was performed at 13 DOF (data not visualized, see Supplementary File S4). There was a 6% reduction in the median prevalence of resistance to 15-membered ring macrolides at the same time point owing to this decrease in use (40.0% vs. 34.2% in the control and treatment experiments, respectively). Decreases in 15-membered ring macrolide use were offset by increases in the median uses of cephalosporins, phenicols, and 16-membered ring macrolides by 50 DOF (n = 524, 149, and 91 uses, respectively). There was no corresponding increase in the median prevalence of resistance at 50 DOF for cephalosporins or phenicols, likely owing to the low mean probability of selection for these classes (calibrated in [27]). There was a very modest increase (1.5%) in the median prevalence of resistance for 16-membered ring macrolides at 50 DOF (16.3% vs. 17.9% in the control and treatment experiments, respectively). The mean probability of selection for this class is higher than for any other calibrated class (see Supplementary File S1).

**Figure 9:**
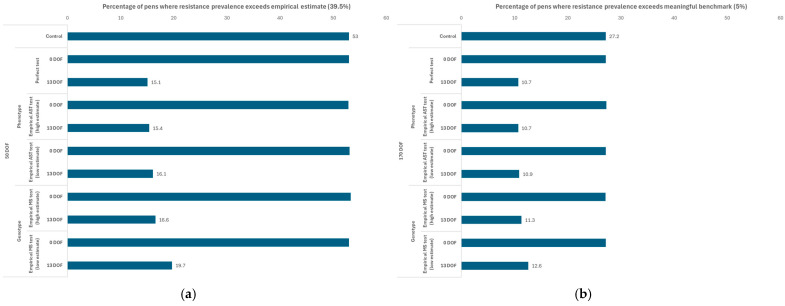
Percentage of *pens* over n = 5000 realizations where the prevalence of resistance to 15-membered ring macrolides exceeded the benchmark value at (**a**) 50 DOF, left and (**b**) 170 DOF, right, for different testing strategies using the “extreme macrolide use” BRD treatment protocol (Figure 2). Empirical sensitivity and specificity values for the antimicrobial susceptibility (AST) and metagenomic sequencing (MS) tests are in Table 4. The benchmarks selected to highlight changes in the distributions of resistance prevalence by antimicrobial class are available in Table 5. Data labels are omitted for TI scenarios with tests performed at 0 DOF, as the values did not differ from the TO control.

The percentage of *pens* over n = 5000 realizations where the prevalence of resistance to 15-membered ring macrolides at 50 and 170 DOF exceeded the benchmark value (Table 5) are reported for the TI scenarios using the “extreme macrolide use” protocol in Figure 9a and Figure 9b, respectively. When the test was performed at 0 DOF, there was no impact of the testing-informed treatment strategy on the distribution of pen-level resistance prevalence at either time point (i.e., the percentage of pens where resistance to 15-membered ring macrolides exceeded the benchmark value was equal to that of the TO control at 50 DOF (53%) and 170 DOF (27%)). When the test was performed at 13 DOF, we observed reductions in the percentage of *pens* where resistance exceeded the benchmark value (i.e., there were fewer pens where resistance met or exceeded the empirically derived benchmark at 50 DOF and the risk-based benchmark at 170 DOF). Less sensitive tests were associated with more modest reductions in the percentage of pens exceeding these benchmark values. For example, when the MS test with the “low” empirical sensitivity estimate (56%) was used, approximately 20% of pens exceeded the benchmark resistance value at 50 DOF; conversely, only 15% of pens exceeded that value when the AST with the “high” empirical sensitivity estimate (86%) was used. By 170 DOF, the percentage of pens exceeding the benchmark value were 13% and 11% for the low sensitivity MS test and high sensitivity AST, respectively (i.e., the impact of test sensitivity on this output was decreased relative to 50 DOF).

Table 6 summarizes the impact of different testing-informed treatment strategies on the median numbers of BRD relapses, BRD deaths and finished cattle by type. As with the other outputs of interest, there was no impact of the strategy on these and other disease and economic outcomes when the test occurred at 0 DOF. When a perfect test was performed at 13 DOF, there were n = 188, 135 and 90 fewer median first, second and third (chronic) BRD relapses related to AMR-linked treatment failure, respectively, relative to the TO control. Because there were fewer cattle removed to the chronic pen with BRD, the median number of healthy cattle finished to the target weight increased by approximately 85 animals. Relatedly, the median number of chronically ill cattle that were euthanized or slaughtered at a reduced weight was less than half of its previous value. Less sensitive tests used at 13 DOF were associated with more modest (1) reductions in the median number of BRD relapses and (2) increases in the median number of healthy finished cattle (i.e., compared to when a perfect test is used).

## 4. Discussion

Feedlot cattle are typically managed as groups at the *pen* level, and emerging research highlights the potential impact of pen-cohort health on individual calves. Horizontal transmission of BRD between pen-mates has been demonstrated [57], and evidence for the “contagious spread” of resistant *M. haemolytica* isolates has been described in both Canadian [32] and American [58] feedlots. Abi Younes and colleagues reported that a calf’s risk of BRD and susceptibility to antimicrobials at the time of treatment were influenced by the pen-level prevalence of BRD-associated bacteria and AMR in [24]; the authors discussed how pen-level sampling strategies might therefore be used to inform AMU protocols in support of antimicrobial stewardship goals. Indeed, Otto et al. [17] expound on the “potential use of laboratory testing [for AMR] at the animal group level” in their application of value stream mapping to the problem of BRD in commercial feedlots. The Pan-Canadian Framework for Action to tackle AMR/AMU notes the importance of research to understand the implications for livestock production of interventions designed to limit AMR risk [59]. As a complement to observational studies [24], simulation studies are ideally suited to experimenting with novel approaches to problems in complex biological systems. In particular, ABMs have the flexibility to incorporate behavioral units at multiple scales in a nested structure (e.g., individual animals within feedlot pens) [28].

An updated version of our recently published feedlot simulation tool [27] was used in this study to examine if and under what conditions a laboratory testing-informed BRD treatment strategy at the *pen* level could meaningfully impact select antimicrobial stewardship and feedlot economic outcomes. We hypothesized that particular combinations of pen sampling schemes and AMR diagnostic tools to guide treatment choice could reduce BRD relapses (i.e., AMR-linked therapeutic failures), total AMU and resultant AMR. A comparison of potential strategies was made possible by the newly incorporated DES workflow, which simulated the sampling and testing of individual *Pen* agents under specified conditions. DES models are more typically associated with efforts to optimize supply chains or processes [26] and have been used to explore strategies for reducing emergency department wait times [60] and other complex problems in human healthcare systems [61]. To our knowledge, this work represents a novel application of a hybrid ABM and DES to the problem of AMU and AMR in the context of BRD management in North American feedlots. Individual-based models have been used in similar contexts to examine the impact of farming practices on BRD dynamics in French fattening farms [62,63]. Specifically, these authors examined factors like pen size and allocation on the spread of BRD-linked pathogens [62] and BRD outcomes [63] in calves of varying risk.

This study investigated the test-only (control) and testing-informed treatment (intervention) variations of 26 unique sampling and AMR testing scenarios (n = 52 total experiments). Several of the parameters related to the *Testing* process and examined here were purposively selected to reflect empirical evidence generated by our research group [17,23,24,27,31,32,43,51] and/or the broader goals for this project [64]. For example, Abi Younes et al. [23] determined via simulation modeling that evidence-based laboratory data on individual pens could be generated by sampling a subset of 20 to 30 animals per pen of 200 calves. By default, this series of experiments thus sampled 20 animals per pen; the number of sampled animals was increased to 40 per pen in targeted sensitivity analyses. The authors in [23] noted that an understanding of bacterial dynamics and antimicrobial susceptibility changes in the early feeding period was required to identify optimal sampling times, a theme mirrored in related works [17,24]. Sampling at 0 DOF (feedlot arrival) is both logistically convenient and provides information about AMR in incoming cattle prior to metaphylaxis [36] and the transmission of BRD pathogens between commingled pen-mates. In contrast, sampling at 13 DOF coincides with the maximum post-metaphylactic interval for tulathromycin [39] (i.e., before the expected peak of first BRD cases) and provides information about AMR after the potential for AMU-linked selection and contagious spread of resistant BRD pathogens [32,58]. Importantly, the *Testing* agent and related infrastructure was constructed such that future users can experiment with different sample size and timing parameters that correspond to their research priorities.

Otto et al. [17] describe a “future state” wherein laboratory data for AMR detection is compiled at the *pen* and *feedlot* levels and analyzed to inform the appropriateness of current BRD treatment plans. In this study, simulated outputs for the count of tests that were positive for resistance by antimicrobial class were generated for both levels of aggregation (see Section 2.7); the test positivity percentages were calculated in a subsequent step and compared across levels. In general, the median AMR test positivity percentage across n = 5000 realizations is similar for both *pens* and *feedlots*, but substantially more variation in this outcome exists at the *pen* level. Our previous modeling study [27] reported on the range of simulated resistance prevalences at each level and discussed the vulnerability of smaller units to the impact of chance events. Aggregate unit-level differences in the true AMR prevalence (reflected in the width of prediction intervals in [27]) were magnified here by the random sampling of a subset of animals as part of the testing process. These observations underscore how our selection of the *pen* as the unit of intervention (i.e., treatment changes based on the tested pen-level prevalence of resistance) could meaningfully impact key outputs of interest. Specifically, a strategy that uses *pen* rather than *feedlot*-level data to inform pen-specific treatment plans (as in this study) will more often trigger changes in the AMU profile owing to the wider distributions of AMR test positivity.

### Effectiveness of Testing-Informed Treatment in the Modern Feedlot Setting

There was no impact of the pen-level testing-informed treatment strategy on the AMU, AMR, or disease outcomes of interest under the baseline conditions (i.e., when the probabilities of resistance on arrival matched those in the reference data set in [27], and when the default BRD treatment protocol was consistent with current management practices). This remained true even when the pen-level resistance prevalence threshold that triggered a “treatment change” was reduced from 25% to 10% in the sensitivity analyses for these scenarios. The probability of resistance at arrival to phenicols, fluoroquinolones, and potentiated sulfonamides (i.e., the default drug classes used to treat first, second, and third BRD cases) was low (<1%) in the empirical data used to calibrate the model in [27]. More recent surveillance data (2022–2024) from CFAASP (not used in model calibration) confirms that resistance to these antimicrobial classes in *M. haemolytica* isolates from calves is negligible at arrival (0%) and remains low through 13 DOF [12,47,54]. Given the low prevalence of resistance to the BRD treatment classes in the sentinel pathogen, the availability of pen-level AMR testing results for this organism to inform therapeutic AMU is unlikely to be effective in advancing antimicrobial stewardship goals in present-day commercial feedlots. The remaining scenarios in this work were curated to maximize responsiveness to AMR and therefore better investigate (1) the ecological and regulatory conditions under which this strategy might be effective, and (2) the impact of imperfect diagnostic tests on the strategy’s potential effectiveness.

The on-arrival probabilities of resistance for *M. haemolytica* isolates were increased to “worst-case” levels in a subset of experiments designed to generate higher prevalences of pen-level resistance to the therapeutic options at 0 and 13 DOF. A scenario of this type could theoretically derive from the increased use of antimicrobials in earlier phases of the beef production chain prior to feedlot entry (e.g., on cow-calf or backgrounding operations). While Fossen et al. [65] reported that AMU patterns in cow-calf herds remained relatively unchanged between 2014 and 2020, the percentage of herds in western Canada using macrolides had significantly increased in the previous 5 years. Further, phenicols (the default drug class for first BRD cases in the expert-developed BRD protocol) were used in 73% of sampled herds, and was the most frequently used drug class to treat respiratory disease in nursing calves [65]. In this work, there were more metaphylactic failures and therefore first BRD cases when the probability of 15-membered ring macrolide resistance was increased at arrival (i.e., relative to the baseline value), but increases in the tested resistance to BRD treatment classes still failed to reach the “treatment change” threshold (25%) at either time point in over 95% of replications. Changes in the distribution of antimicrobial uses by class were observed when the threshold was reduced to 10%, but these were not associated with reductions in AMR prevalence or BRD relapses.

*M. haemolytica* is often regarded as the primary bacterial pathogen associated with acute BRD [66], and this organism serves as the sentinel pathogen in this and our previous modeling study [27]. It follows that the diagnostic AMR data being compiled to inform BRD treatment in these experiments is based on a single BRD-associated pathogen [21,67], which we assumed here to be representative of clinically relevant AMR in the nasopharynx of beef cattle. Emerging surveillance data from Canadian feedlots suggests that a greater proportion of *P. multocida* and *H. somni* than *M. haemolytica* isolates are resistant to one or more tested antimicrobials at both arrival and later in the feeding period [12,54]. In the 2024 surveillance year, the population-averaged prevalences of resistance to florfenicol (used in this model for first BRD cases) and enrofloxacin (used in this model for first BRD relapses) were 9% and 11% in *P. multocida* isolates from calves at arrival (n = 68), respectively (S. Gow, personal communication). These values exceed even those used for *M. haemolytica* in the exploratory “worst-case” experiments in this study. *P. multocida* was the most prevalent BRD-associated bacteria in the upper respiratory tract of healthy western Canadian beef calves in a recent study [68]; likewise, it was the most frequently isolated bacteria in the lower respiratory tracts of cattle with BRD in [35]. The availability of pen-level AMR testing results that account for the polymicrobial nature of BRD might thus be more effective in reducing resistance-linked treatment failures and related AMU. Calibration of this model to temporal resistance prevalence data for other BRD pathogens is increasingly possible with the recent availability of published data [12,47,54].

The potential effectiveness of the laboratory testing-informed treatment strategy in these simulations is critically dependent on the selection of the “user-defined pen-level AMR threshold” [17]. Intervention-associated changes in the disease, treatment and resistance outcomes of interest (i.e., demonstrable “effectiveness”) are only possible if the pen-level prevalence of resistance to the first-line drug meets the “treatment change” threshold. The default threshold in this work (25%) was purposefully selected to exceed the empirically derived probability of all-cause treatment failure for first BRD cases (21.6%) in the initial model [27] (see Section 2.3.5) and was the same for all antimicrobial classes. Indeed, Lubbers and Turnidge opine in [42] that “other first-line therapies may need to be considered when the percentage of BRD pathogens classified as resistant to any one antimicrobial agent is >25%”. However, the selection of pen-level AMR thresholds might reasonably reflect other regulatory or proprietary (e.g., economic or management) priorities; Otto et al. [17] describe the potential for veterinary practices to customize these thresholds in their feedlot management software. For example, it might be prudent to establish different “treatment change” thresholds that account for the relative importance of the antimicrobial class to human medicine [55] (e.g., comparatively lower thresholds for Category I drugs of very high importance, including cephalosporins and fluoroquinolones). Alternatively, efforts to limit the *new* emergence of AMR in feedlot settings could be supported by establishing thresholds at historical levels of antimicrobial resistance (e.g., [33]). Careful consideration should be given to the balance of “treatment change” thresholds across the classes of antimicrobials used for BRD treatment, given the risk trade-offs (e.g., increased use of critically important antimicrobials or antimicrobials with higher selective potential).

#### Theoretical Applications of the Testing-Informed Treatment Strategy

In a different subset of thought experiments designed to maximize selection pressure for AMR, the baseline (i.e., feedlot standard) BRD treatment protocol was replaced with a theoretical “extreme 15-membered ring macrolide use” protocol involving repeated exposures to a single antimicrobial class. It is generally recommended that antimicrobials from the same class should not be used repeatedly for both control and treatment of BRD [35,69]; in their review of alternative practices to AMU in feedlots, the National Collaborating Centre for Infectious Diseases notes that many feedlots employ “antimicrobial rotational strategies” wherein calves that develop pneumonia after receiving a metaphylactic drug will be treated with a different, unrelated antimicrobial [70]. Nevertheless, practical and cost-related reasons for prescribing the same drug or drug class for metaphylaxis and BRD treatment have been described [71]. Further, domestic or international regulations that restrict the diversity of drugs available for use in food-producing animals may limit the opportunity to “cycle” antimicrobial classes, making repeated exposures more likely. The Canadian Academy of Health Sciences recently described five strategic interventions with promise for strengthening antimicrobial stewardship in food-producing animals [72]; among these is the requirement that the therapeutic use of Category I antimicrobials in farmed animals be justified with laboratory evidence, as was adopted in Quebec. While the “extreme” treatment protocols used in these experiments remain theoretical, they allowed us to more fully explore how (1) sample timing and (2) diagnostic test accuracy affect the success of this strategy.

The availability of pen-level AMR testing results from samples at arrival (0 DOF) to inform BRD treatment had no effect on the stewardship or economic outcomes of interest for any scenario in this study. This remained true even for experiments using the aforementioned “extreme” protocol, where the treatment options were intentionally selected to maximize potential impacts. Samples from this time point failed to capture the significant shifts in respiratory microflora [73] and antimicrobial susceptibility [74,75] linked to the administration of metaphylactic antimicrobials; specifically, the near-term increases in resistance were not reflected in the diagnostic test information, resulting in AMR-linked treatment failures. Conversely, improvements in key stewardship (e.g., fewer total antimicrobial uses) and economic (e.g., more healthy cattle fed to target weight) outputs were observed when pen-level testing information from samples at 13 DOF were available to inform treatment in the “extreme” protocol experiments. This is broadly consistent with the suggestion in [24] that AMR outcomes from cattle near two weeks on feed can inform “antimicrobial susceptibility results at [the] time of first BRD treatment”. Importantly, most BRD cases are expected to occur after 13 DOF in high-risk calves that receive metaphylactic tulathromycin [24,40]; in the empirical BRD incidence data used to parameterize this model, only 20% of the animals that will get sick have a first case of BRD prior to 13 DOF [27]. This is nevertheless an important limitation of our assumption that pen-level AMR can be determined in advance of the need to treat for BRD, given that test results from 13 DOF were unavailable for a minority proportion of cases.

Unlike sampling at 0 DOF, which would coincide with routine cattle processing at feedlot entry, sampling at 13 DOF would require a new, added process that involves “collecting samples from [calves] in the feedlot handling facility outside of typical animal handling” [17]. The authors in [17,76] detail the substantial financial and human resource costs associated with the implementation of the proposed strategy, and note that its uptake by feedlots would require it demonstrated benefit in terms of animal health (e.g., fewer treatment failures) and production economics (e.g., reduced treatment costs). Given the theoretical/exploratory nature of the “extreme” protocol experiments in this study, a more thorough economic analysis using the generated outputs would not have been instructive and was not completed here. However, a future cost–benefit analysis would need to account for the novel costs associated with sample collection, shipping and laboratory testing with the appropriate test [76,77]; consideration should be given to (1) the number of samples per pen that are sufficient to inform treatment [23], and (2) the delay in time to results that might limit the utility of testing information [17,22,76,77]. For example, while fewer samples (e.g., 20 vs. 40) may reduce the financial and logistical burden of the testing strategy, related imprecision in the tested pen-level AMR estimate could lead to unnecessary (and potentially costly) treatment changes. Further, existing test options are limited in their ability to provide timely results [22,77], with implications for the efficiency of testing costs. Delays for sample shipping (1.5 days) and diagnostic testing (3 days) were incorporated into the *Testing* agent in this study; close to 30% of the animals that got sick had a first case of BRD before the test results were available at 17 DOF.

As an indicator of “information quality” with respect to the proposed testing strategy, the authors in [17] query how well the resulting test information reflects the *true* antimicrobial susceptibility of the BRD pathogens. Owing to the recent availability of empirical estimates for the sample-level sensitivity and specificity of candidate diagnostic tests [43], this study compared the relative performance of these imperfect alternatives to a hypothetically “perfect” standard. Of specific interest was the impact of variable test accuracy on (1) the distributions of AMR test positivity values in relation to the “treatment change” threshold, and (2) the relative changes in median numbers of BRD events (relapses, deaths) and BRD-related chronic pen usage. A highly sensitive test is generally more important than a highly specific test when the prevalence of a condition is high [78], as was the case with 15-membered ring macrolide resistance in *M. haemolytica* isolates at 13 DOF [27]. In the “extreme” protocol experiments, the theoretical improvements in target outcomes associated with the pen-level testing strategy (i.e., when a perfect test is performed) were reduced in a step-wise fashion that corresponded to decreasing empirical test sensitivity. It is notable that these reductions were nevertheless moderate relative to the sizeable decreases in test sensitivity (i.e., favorable changes in key outcomes were largely retained with the less sensitive tests). For example, there were only 11% more antimicrobial uses and 7% more BRD relapses when the MS test with the “low” empirical sensitivity estimate (56%) was used compared to when the counterfactual “perfect” standard was applied. This suggests that the information generated by currently available but imperfect diagnostic tests [77] may be sufficient to inform treatment under certain conditions.

A highly specific test is generally more important than a highly sensitive test when the prevalence of a condition is low [78], as was the case with resistance to the BRD treatment classes in this study’s experiments (≤5%) [27]. In the “high incoming AMR” experiments, the information from the more sensitive but less specific MS test used at either time point triggered unnecessary changes to the BRD treatment protocol. The observed increases in Category I AMU (i.e., of ceftiofur, a cephalosporin) did not “buy” fewer BRD relapses or more cattle finished to the target weight in these particular scenarios. Conversely, the failure of the information from the less sensitive but more specific AST at 13 DOF to trigger appropriate changes to the BRD treatment protocol (i.e., when the true pen-level resistance exceeded the 25% “treatment change” threshold) had virtually no impact on the downstream outputs of interest. These observations highlight the extremely complex relationships between the default BRD treatment protocol (often unique to the feeding operation), the dynamic prevalences of resistance to the treatment classes, the accuracy of available diagnostic tests for detecting AMR (which differs by class), and the selected “treatment change” thresholds (which could theoretically differ by class). The successful implementation of a pen-level sampling and diagnostic strategy must necessarily consider the careful balance of these and other interrelated factors; this feedlot simulation tool offers a novel opportunity to experiment with and optimize the strategy under different ecological and management conditions.

Several important limitations of this work deserve mention. Due to the lack of published data, we were unable to curate a longitudinal data set with the time-varying proportion of *M. haemolytica* isolates with known ARGs against which to calibrate a truly “genotypic” version of this model. In order to experiment with MS as a candidate diagnostic test in this study, we assumed that the AMR selection, waning and transmission parameters calibrated in [27] for the “phenotypic” version of this model were suitable estimates for the acquisition and loss of genes that confer resistance to those antimicrobial classes. Plainly, we assumed perfect concordance between the pathogen’s AMR phenotype and genotype; however, it is generally understood that the presence of ARGs does not guarantee phenotypic resistance [49,51]. Previous analyses have described highly variable genotype-phenotype concordance rates for *M. haemolytica* and other BRD pathogens [79]. A more recent effort to annotate known resistance genes in *M. haemolytica* genomes [80] reported >90% concordance between phenotypic resistance and the presence of known ARGs for seven BRD drugs from four antimicrobial classes. It is notable that the inclusion of *estT* [81] in [80], used as the reference gene for 16-ring macrolides in these experiments, improved the concordance rate for tilmicosin in previously sequenced *M. haemolytica* isolates [82]. Calibrating a “genotypic” version of this model remains a distinct possibility with the increasing availability of surveillance and other data [49] with a focus on genetic resistance.

A novel component of this work was its use of empirical estimates for diagnostic test sensitivity and specificity to parameterize the *Testing* agent. In particular, we distinguished between the “phenotypic” and “genotypic” approaches to AMR diagnostics by incorporating antimicrobial class-level test accuracy values derived from field data [43] for AST and MS, respectively. Reliable estimates could not be generated in [43] for classes of antimicrobials with low levels of phenotypic and/or genetic resistance, including many of those used to treat BRD in the expert-developed protocol. Conservative placeholder values were adopted for these drugs/genes in the experiments that used empirical (rather than perfect) tests, and the resultant outputs are therefore best interpreted as general trends rather than precise insights. It was similarly difficult to calibrate the model in [27] to reference data for the antimicrobial classes with near-zero (<1%) prevalences of resistance over the feeding period, and our confidence in the calibrated parameter values for these classes (e.g., phenicols, cephalosporins) remains low. As with all models, the quality of the simulated output is directly related to the quality of the information received as input. In combination with our previous work [27], this study demonstrates how the feedlot model can be used to experiment with interventions proposed to limit AMR risk in the context of BRD management. Importantly, the model can readily accommodate updates to its inputs or infrastructure to best reflect the data emerging from this dynamic field of research.

## Figures and Tables

**Table 1:** Fixed treatment protocols for non-BRD prophylactic and therapeutic indications in this series of experiments. Protocols were selected probabilistically from the options detailed in [27] and held constant across the experiments in this study.

Antimicrobial Use Type	Indication	Condition, If Applicable	Case Description	Default Antimicrobial Selection(s)	Regimen
Prophylaxis (in-feed at *pen* level)	Histophilosis	--	--	Chlortetracycline	2 × 5-day courses of high-dose CTC ^1^ (18–23 DOF, 25–30 DOF)
	Liver abscesses	--	--	Chlortetracycline Tylosin	Low-dose CTC starts at 42 DOF; switch to TYL ^2^ 28 days before end of feeding period
	Foot rot	Detected in 10% of animals in same pen (cumulative)	--	Chlortetracycline	7-day course of high-dose CTC
Treatment (injectable at *calf* level)	Arthritis	Lightweight at time of detection (<1000 lbs)	First case/pull	Oxytetracycline	3 doses (single dose every 3 days)
			First relapse (second case/pull)	Trimethoprim–sulfadoxine	5 doses (single dose per day × 5 days)
			Second relapse (third case/pull)	Ceftiofur CFA ^3^	2 doses (single dose every 4 days)
		Heavyweight at time of detection (>1000 lbs); and selected for treatment (50%)	First or subsequent case/pull	Ceftiofur HCl ^3^	Single dose
	Foot rot	Lightweight at time of detection (<1200 lbs)	First or subsequent case/pull	Penicillin G	Single dose
		Heavyweight at time of detection (>1200 lbs)	First or subsequent case/pull	Ceftiofur HCl	Single dose

^1^ CTC is the abbreviation for chlortetracycline. ^2^ TYL is the abbreviation for tylosin. ^3^ Ceftiofur is not expected to be effective against uncomplicated *Mycoplasmopsis bovis*-associated arthritis [41]. However, arthritis diagnosed late in the feeding period in fall-placed calves can be confounded by other infectious agents, and some cases are likely sequelae of chronic and unresponsive foot rot.

**Table 2:** New and/or updated values and sources for parameters in the agent-based feedlot model.

Parameter	Condition	Value in Baseline Model	Source or Rationale, If Applicable
***Cattle agent parameters***			
Average daily gain (ADG) for healthy steer calves	*Applies to steers with no BRD or arthritis history that were lighter-weight on arrival*	Selected from normal distribution with µ = 3.50 and σ = 0.44 pounds/day	Empirical data from 7685 steer calves with arrival weights ranging from 500–799 pounds (2019–2023)
Absolute decrease in ADG for animals with first case of BRD	*Applies for remainder of feeding period to animals with single diagnosis* (i.e., *first case*) *of BRD*	0.0453 pounds/day (relative to healthy animals)	Empirical data from 1630 steer calves with arrival weights ranging from 500–799 pounds (2019–2023)
Absolute decrease in ADG for animals with first relapse of BRD	*Applies for remainder of feeding period to animals with first relapse* (i.e., *second case*) *of BRD*	0.1759 pounds/day (relative to healthy animals)	Empirical data from 366 steer calves with arrival weights ranging from 500–799 pounds (2019–2023)
Absolute decrease in ADG for animals with second relapse of BRD	*Applies for remainder of feeding period to animals with second or more relapses* (i.e., *third or subsequent cases*) *of BRD*	0.3407 pounds/day (relative to healthy animals)	Empirical data from 162 steer calves with arrival weights ranging from 500–799 pounds (2019–2023)
Absolute decrease in ADG for animals with first case of arthritis	*Applies for remainder of feeding period to animals with single diagnosis* (i.e., *first case*) *of arthritis*	0.2538 pounds/day (relative to healthy animals)	Empirical data from 131 steer calves with arrival weights ranging from 500–799 pounds (2019–2023)
Absolute decrease in ADG for animals with first relapse of arthritis	*Applies for remainder of feeding period to animals with first relapse* (i.e., *second case*) *of arthritis*	0.4625 pounds/day (relative to healthy animals)	Empirical data from 32 steer calves with arrival weights ranging from 500–799 pounds (2019–2023)
Absolute decrease in ADG for animals with second or third relapse of arthritis	*Applies for remainder of feeding period to animals with second or more relapses* (i.e., *third or subsequent cases*) *of arthritis*	0.8778 pounds/day (relative to healthy animals)	Empirical data from 3 steer calves with arrival weights ranging from 500–799 pounds (2019–2023)
***Testing agent parameters***			
Number of animals sampled per single pen	*Applies to all home pens in the feedlot (not hospital or chronic pens)*	20 *	Simulated data from [23]
Time delay for collection of single nasopharyngeal sample	*Delay applies to both diagnostic test types (AST and metagenomic sequencing)*	1 min	Observations from sample collection step for multi-year research [32] and CFAASP projects [37,47,48]
Time delay for transport of nasopharyngeal sample to diagnostic laboratory	*Delay applies to both diagnostic test types (AST and metagenomic sequencing)*	36 h	Observations from streamlined sample transport step for multi-year CFAASP project [37,47,48]
Time delay for nasopharyngeal sample processing and diagnostic testing	*Delay applies to both diagnostic test types (AST and metagenomic sequencing)*	72 h	Observations from sample processing for multi-year research [31,32] and CFAASP [37,47,48] projects
Time delay for reporting of diagnostic result to feedlot veterinarian	*Delay applies to both diagnostic test types (AST and metagenomic sequencing)*	0 min (instantaneous)	Model parsimony
Threshold at which test result for pen-level AMR triggers a change in BRD treatment	*Applies to all antimicrobial drug classes examined in* *the model*	25% *	Empirical data reported in [27], expert opinion [42]

* Model assumptions explored in sensitivity analyses in this study.

**Table 3:** Values used in the experiments for the on-arrival prevalence of resistance to select antimicrobial drugs in *Mannheimia haemolytica* in the (a) baseline and (b) high on-arrival AMR scenarios.

		Probability of Resistance at Arrival (CI)	
Antimicrobial Class	Reference Drug ^1^	(a) Baseline Scenarios ^2^	(b) High (Worst-Case) Scenarios ^3^
Cephalosporins	Ceftiofur	0% (0%, 100%)	5.1% (0%, 5.1%)
Fluoroquinolones	Enrofloxacin	0.4% (0.2%, 0.9%)	5.1% (0.2%, 5.1%)
15-membered ring macrolides	Tulathromycin	2.4% (1.8%, 3.3%)	9.3% (1.8%, 9.3%)
16-membered ring macrolides	Tilmicosin	4.3% (3.4%, 5.4%)	5.1% (3.4%, 5.1%)
Potentiated sulfonamides	Trimethoprim	0.3% (0.1%, 0.6%)	5.1% (0.1%, 5.1%)
	Sulfadimethoxine	4.3% (3.5%, 5.3%)	74.8% (3.5%, 74.8%)
Phenicols	Florfenicol	0.1% (0.03%, 0.5%)	5.1% (0.03%, 5.1%)
Tetracyclines	Oxytetracycline	4.9% (4.1%, 5.7%)	9.3% (4.1%, 9.3%)

^1^ The antimicrobial drug with the most complete data set (i.e., one or more raw data points for each time point) was selected to represent all others in its class in the calibration experiments in [27]. ^2^ In the experiments with **baseline on-arrival resistance,** the estimated probability of phenotypic resistance to each antimicrobial at arrival was equal to the values used in the calibration and Monte Carlo experiments in [27]. ^3^ In the experiments with **high (worst-case) on-arrival resistance,** the probability of phenotypic resistance to each antimicrobial at arrival was equal to the upper confidence interval of the estimated prevalence for feedlot calves sampled in 2022 as part of CFAASP [12,47].

**Table 5:** Percentages selected as meaningful benchmarks to highlight changes in the distributions of resistance prevalence by antimicrobial class at select time points across different sampling and testing scenarios. The proportion of realizations (n = 5000) where the prevalence of resistance exceeded the benchmark percentage was calculated in a subsequent step for both the *pen* and *feedlot* levels. The proportions are reported as summary outputs in the data repositories (Supplementary Files S3 and S4).

Antimicrobial Category ^1^	Antimicrobial Class	Reference Drug	Benchmark Percentage at 50 and 70 DOF ^2^	Benchmark Percentage at 170 DOF ^3^
Category I	Cephalosporins	Ceftiofur	0%	0%
	Fluoroquinolones	Enrofloxacin	0%	0%
Category II	15-membered ring macrolides	Tulathromycin	39.5%	5%
	16-membered ring macrolides	Tilmicosin	37.5%	5%
	Potentiated sulfonamides	Trimethoprim	1.6%	5%
		Sulfadimethoxine	51.1%	5%
Category III	Phenicols	Florfenicol	0%	10%
	Tetracyclines	Oxytetracycline	34.3%	10%

^1^ Category refers to the ranking of antimicrobial drugs based on their importance to human medicine as determined by Health Canada [55]. ^2^ Benchmark values equal to the estimated prevalence of antimicrobial resistance in *M. haemolytica* isolates (n = 69) from feedlot calves sampled at rehandling in 2023 as part of CFAASP [12,54]. ^3^ Benchmark values intentionally selected to reflect the relative importance of the drug to human medicine and the concern posed by resistance in the late feeding period [55]. Values were informed by levels of resistance as defined by the European Food Safety Authority [56], where <0.1% resistance is “rare”, and 0.1–10% resistance is “very low” or “low”.

**Table 6:** Impact of different testing-informed treatment strategies on the (1) change in median number of total drug uses for treating BRD; (2) median number of BRD relapses and deaths; and (3) median number of finished cattle by health status and weight for scenarios using the “extreme 15-membered ring macrolide use” protocol (Figure 2b).

Test at	Diagnostic Paradigm	Scenario	Change in Median Number Drug Uses	Median Number BRD ^1^				Median Number Cattle at End ^2^			
				First Relapse	Second Relapse	Chronic Cases	Deaths	Healthy (Target Weight)	Chronic (Target Weight)	Chronic (Reduced Weight)	Euthanize
		Control (test-only)	--	492	267	141	70	9236	45	46	47
0 DOF	Phenotype	Perfect test	0	492	267	141	70	9236	45	46	47
		Empirical AST (high estimate)	0	492	267	141	70	9236	45	46	47
		Empirical AST (low estimate)	0	493	267	140	70	9236	45	46	47
	Genotype	Empirical MS (high estimate)	0	493	267	141	70	9235	45	46	48
		Empirical MS (low estimate)	0	493	267	141	70	9236	44	46	48
13 DOF	Phenotype	Perfect test	−322	304	132	51	72	9320	17	17	18
		Empirical AST (high estimate)	−322	305	133	51	72	9320	17	17	18
		Empirical AST (low estimate)	−319	308	135	53	73	9318	17	18	18
	Genotype	Empirical MS (high estimate)	−313	312	138	54	72	9317	18	18	19
		Empirical MS (low estimate)	−288	325	148	61	72	9310	20	21	21

^1^ There were 9600 fall-placed steer calves placed in 48 “home pens” in the simulation feedlot (i.e., 200 calves per pen). When the “extreme macrolide use” protocol was used, there were 929 first cases of BRD in the “test-only” control (9.7% of animals entering the feedlot). By 170 DOF, there were 1666 total drug uses to treat BRD cases in the “test only” experiment. ^2^ The total number of chronic (target weight), chronic (reduced weight) and euthanized animals at simulation end includes both BRD-affected and arthritis-affected calves and is therefore not expected to equal the number of chronic BRD cases.

## Data Availability

The data sets described in this article were included as Supplementary Files.

## Supplementary Materials

The following supporting information can be downloaded at: https://www.mdpi.com/article/10.3390/antibiotics14101009/s1, Supplementary File S1: ODD Protocol: Virtual Feedlot Model. This file provides complete protocol documentation for the underlying simulation model. The full details on the model structure and parameters are provided here, while innovations made to this model for this paper are highlighted in the main text; Supplementary File S2: Supplementary tables and figures. Contains figures showing evaluation of model performance for each antimicrobial class against independent surveillance data as well as a table with data providing verification that model outputs were consistent with empirically-driven model inputs for the base scenarios; Supplementary File S3: MC Output Data—Excel file with baseline BRD treatment protocol experiments; Supplementary File S4: MC Output Data—Excel file with extreme BRD treatment protocol experiments.

## Abbreviations

The following abbreviations are used in this manuscript:

ABM	Agent-based Model
ADG	Average Daily Growth
AMR	Antimicrobial Resistance
AMU	Antimicrobial Use
ARG	Antimicrobial Resistance Gene
AST	Antimicrobial Susceptibility Testing
BLCM	Bayesian Latent Class Model
BRD	Bovine Respiratory Disease
CFAASP	Canadian Feedlot Antimicrobial Use and Antimicrobial Resistance Surveillance Program
CI	Confidence Interval
CrI	Credible Interval
DES	Discrete Event Simulation
DOF	Days On Feed
IQR	Interquartile Range
LCA	Latent Class Analysis
MS	Metagenomic Sequencing
ODD	Overview, Design Concepts, and Details
TI	Testing Informed
TO	Testing Only
WHO	World Health Organization
