# Supplementary material for: The Potential for Sample Testing at the Pen Level to Inform Prudent Antimicrobial Selection for Bovine Respiratory Disease Treatment: Investigations Using a Feedlot Simulation Tool
Source: Antibiotics (Basel). 2025 Oct 11;14(10):1009. doi: 10.3390/antibiotics14101009 (PMC12562166; doi:10.3390/antibiotics14101009)
Supplement: Supplementary file 1 [file antibiotics-14-01009-s001.zip › Supplement File S2 - Supplemental Figures and Tables.pdf]

The potential for sample testing at the pen level to inform prudent antimicrobial selection for bovine respiratory disease treatment: investigations using a feedlot simulation tool

Dana E. Ramsay, Wade McDonald, Sheryl P. Gow, Lianne McLeod, Simon J.G. Otto, Nathaniel D. Osgood, and Cheryl L. Waldner

Supplementary File S2:
Supplementary figures and tables

Figures S2.1 to S2.5 – Evaluation of model performance for each key antimicrobial class against emerging independent surveillance data.

Table S2.1. Verification that model outputs at the *pen* and *feedlot* levels across n = 5,000 Monte Carlo simulations are consistent with empirically-derived model inputs for the baseline scenario (i.e., test-only control setting).

Supplementary figures and tables

Figure S2.1. Evaluation of model performance for 15-membered ring macrolides against emerging, independent surveillance data. Figure depicts the range of likely resistance prevalence outcomes at the pen (yellow) and feedlot (grey) levels derived from the repeated random sampling of model inputs across 5,000 Monte Carlo simulations (first reported in [27]); in blue is the reference data used to calibrate the model. Superimposed on the figure in black are the population-averaged resistance prevalence estimates (and 95% confidence intervals) for *Mannheimia haemolytica* isolates from calves sampled at rehandling in 2022 (median DOF = 14), 2023 (median DOF = 70), and 2024 (median DOF = 45) as part of CFAASP [12, 47, 54]; these data were *not* used to calibrate the model in [27].

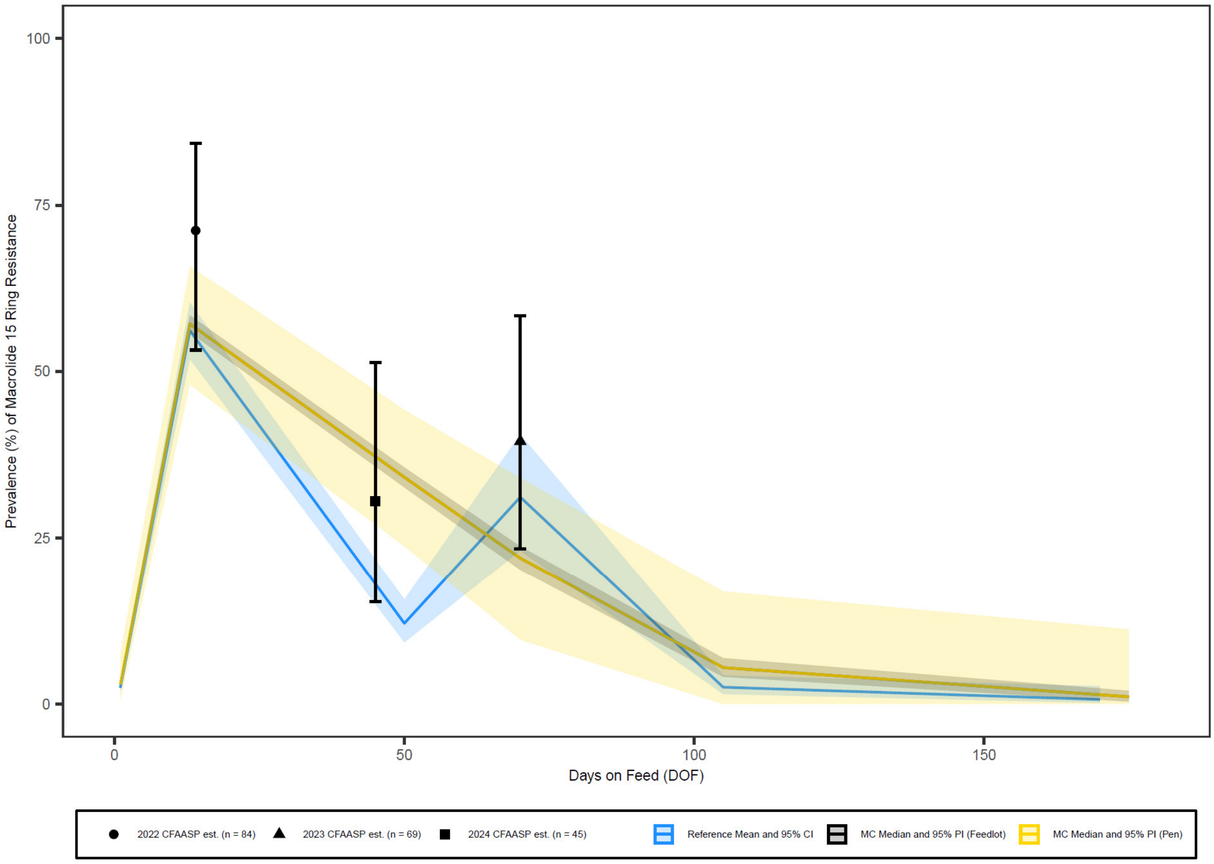

Figure S2.2. Evaluation of model performance for 16-membered ring macrolides against emerging, independent surveillance data. Figure depicts the range of likely resistance prevalence outcomes at the pen (yellow) and feedlot (grey) levels derived from the repeated random sampling of model inputs across 5,000 Monte Carlo simulations (first reported in [27]); in blue is the reference data used to calibrate the model. Superimposed on the figure in black are the population-averaged resistance prevalence estimates (and 95% confidence intervals) for *Mannheimia haemolytica* isolates from calves sampled at rehandling in 2022 (median DOF = 14), 2023 (median DOF = 70), and 2024 (median DOF = 45) as part of CFAASP [12, 47, 54]; these data were *not* used to calibrate the model in [27].

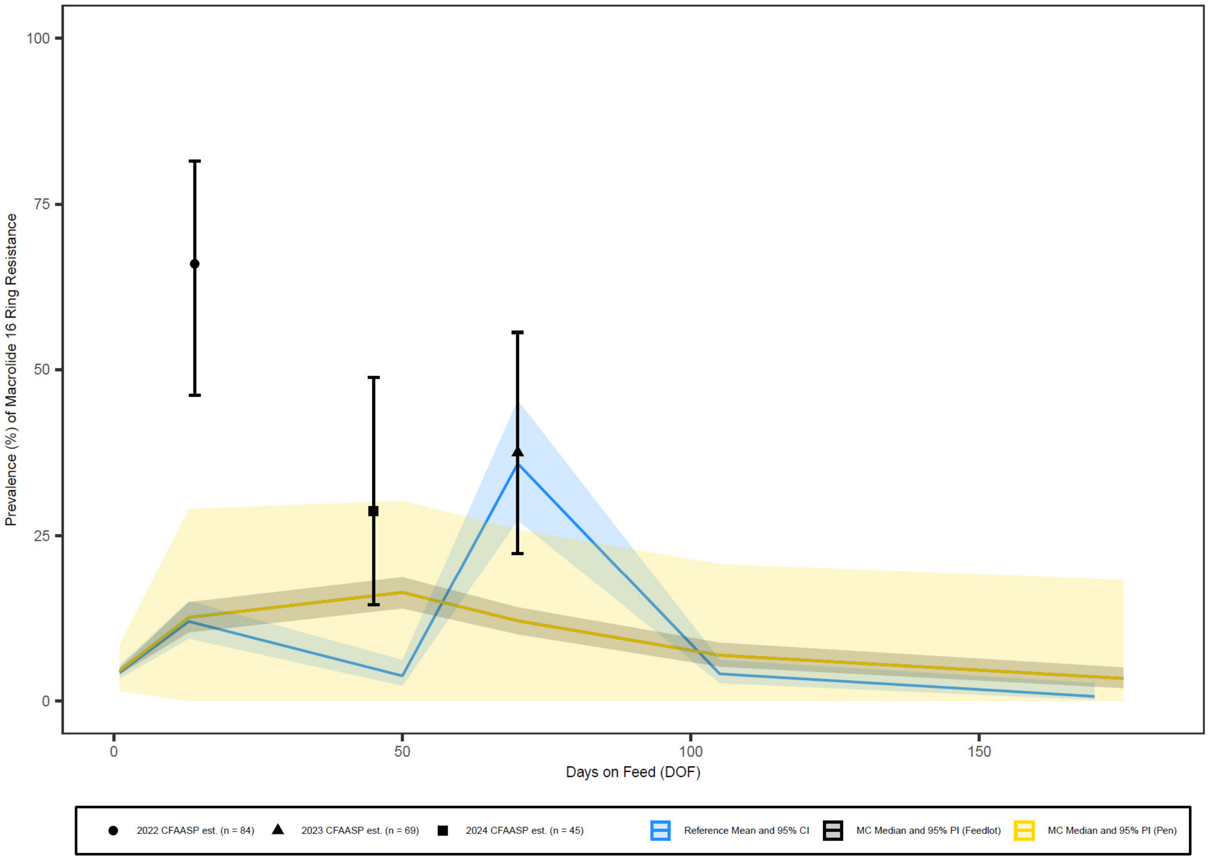

Figure S2.3. Evaluation of model performance for sulfonamides against emerging, independent surveillance data. Figure depicts the range of likely resistance prevalence outcomes at the pen (yellow) and feedlot (grey) levels derived from the repeated random sampling of model inputs across 5,000 Monte Carlo simulations (first reported in [27]); in blue is the reference data used to calibrate the model. Superimposed on the figure in black are the population-averaged resistance prevalence estimates (and 95% confidence intervals) for *Mannheimia haemolytica* isolates from calves sampled at rehandling in 2022 (median DOF = 14), 2023 (median DOF = 70), and 2024 (median DOF = 45) as part of CFAASP [12, 47, 54]; these data were *not* used to calibrate the model in [27].

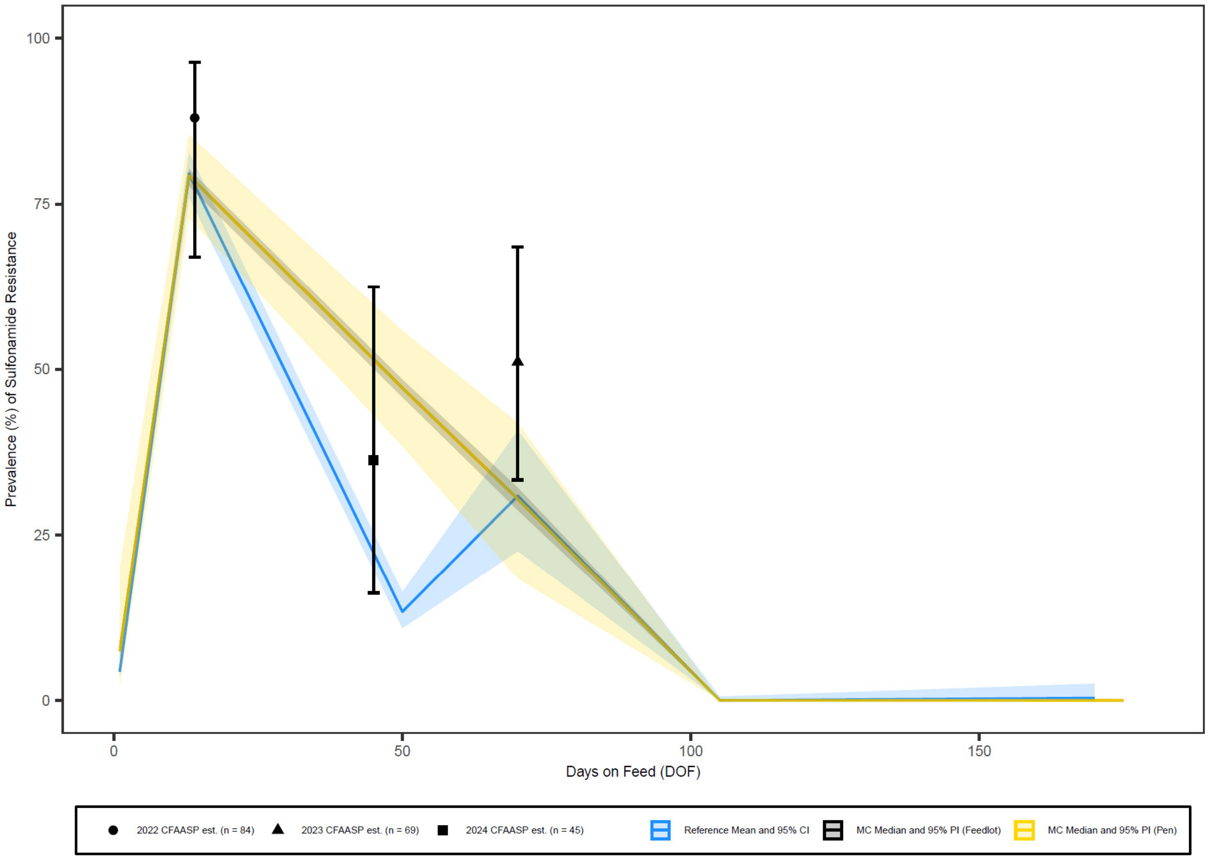

Figure S2.4. Evaluation of model performance for trimethoprim against emerging, independent surveillance data. Figure depicts the range of likely resistance prevalence outcomes at the pen (yellow) and feedlot (grey) levels derived from the repeated random sampling of model inputs across 5,000 Monte Carlo simulations (first reported in [27]); in blue is the reference data used to calibrate the model. Superimposed on the figure in black are the population-averaged resistance prevalence estimates (and 95% confidence intervals) for *Mannheimia haemolytica* isolates from calves sampled at rehandling in 2022 (median DOF = 14), 2023 (median DOF = 70), and 2024 (median DOF = 45) as part of CFAASP [12, 47, 54]; these data were *not* used to calibrate the model in [27].

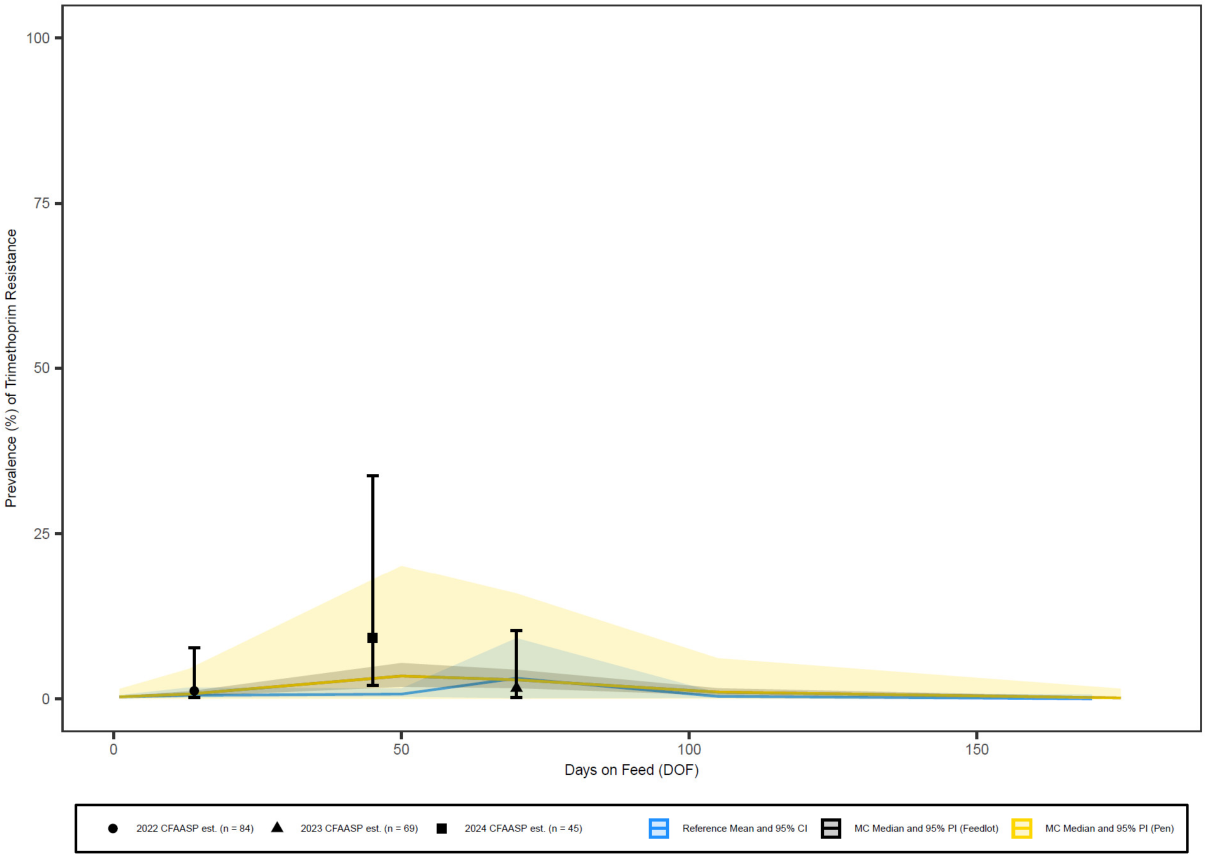

Figure S2.5. Evaluation of model performance for tetracyclines against emerging, independent surveillance data. Figure depicts the range of likely resistance prevalence outcomes at the pen (yellow) and feedlot (grey) levels derived from the repeated random sampling of model inputs across 5,000 Monte Carlo simulations (first reported in [27]); in blue is the reference data used to calibrate the model. Superimposed on the figure in black are the population-averaged resistance prevalence estimates (and 95% confidence intervals) for *Mannheimia haemolytica* isolates from calves sampled at rehandling in 2022 (median DOF = 14), 2023 (median DOF = 70), and 2024 (median DOF = 45) as part of CFAASP [12, 47, 54]; these data were *not* used to calibrate the model in [27].

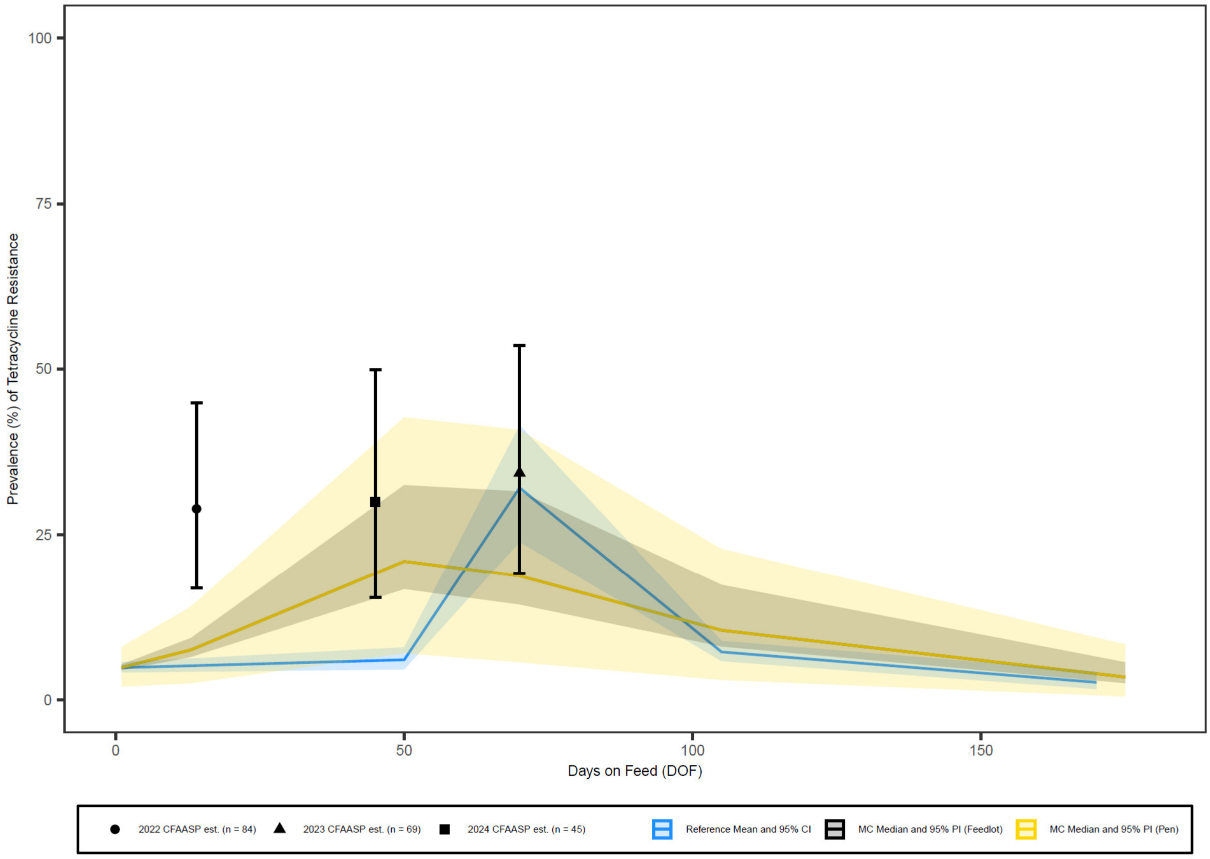

Table S2.1. Verification that model outputs at the *pen* and *feedlot* levels across n = 5,000 Monte Carlo simulations are consistent with empirically-derived model inputs for the baseline scenario (i.e., test-only control setting).

	**Expected value, based on input (if applicable)**	**Observed value**	
		**Pen level (median [95% PI])**	**Feedlot level (median [95% PI])**
Arrival outputs			
Number of calves placed	9600	200 (200, 200)	9600 (9600, 9600)
Average arrival weight (pounds)	500 - 600	550 (546, 554)	549 (549, 550)
Disease outputs			
Number of first BRD cases	9.4% of all calves	--	931 (873, 988)
Number of second BRD cases (first relapses)	21.6% of first cases	--	200 (174, 227)
Number of third BRD cases (second relapses)	35.8% of second cases	--	71 (55, 89)
Number of first arthritis cases	1.0% of all calves	--	94 (75, 113)
Number of second arthritis cases (first relapses)	11.6% of first cases	--	10 (4, 18)
Number of third arthritis cases (second relapses)	20.5% of second cases	--	2 (0, 5)
Number of fourth arthritis cases (third relapses)	19.6% of third cases	--	0 (0, 2)
Number of foot rot cases	3.0% of all calves	--	263 (229, 298)
Specialty pen outputs			
Number of calves with arthritis in hospital pen	All cases <1000 pounds & 50% of cases >1000 pounds	2 (0, 5)	90 (72, 109)
Calf-days in hospital pen	--	26 (0, 74)	1266 (995, 1556)
Number of calves with BRD in chronic pen	35.8% of third cases	0 (0, 2)	25 (16, 36)
Number of calves with arthritis in chronic pen	50% of cases >1000 pounds	0 (0, 1)	4 (1, 9)
Calf-days in chronic pen	--	0 (0, 254)	1982 (1077, 3133)
Mortality outputs			
Number of BRD deaths	8.8% of first BRD cases	1 (0, 4)	73 (57, 91)
Average weight at BRD death (pounds)	--	718 (0, 1197)	804 (757, 856)
Number of histophilosis deaths	0.4% of all calves	1 (0, 3)	35 (24, 46)
Average weight at histophilosis death (pounds)	--	610 (0, 1066)	769 (724, 829)
Number of other-cause deaths	1.3% of all calves	2 (0, 6)	121 (97, 145)
Average weight at other-cause death (pounds)	--	905 (0, 1333)	932 (871, 995)
Economics/finishing outputs			
Number of healthy calves finished to target weight	--	195 (190, 199)	9343 (9309, 9376)
Average finishing weight of healthy calves (pounds)	1325 - 1500	1417 (1332, 1498)	1416 (1332, 1498)
Average daily gain (pounds per day) for healthy calves	3.46	3.48 (3.41, 3.56)	3.48 (3.47, 3.50)
Average days on feed for healthy calves	--	248 (224, 274)	249 (224, 272)
Number of chronic calves finished to target weight	33% of chronic calves	0 (0, 1)	9 (4, 16)
Number of chronic calves finished to reduced weight	33% of chronic calves	0 (0, 1)	9 (4, 16)
Number of chronic calves euthanized	33% of chronic calves	0 (0, 1)	10 (4, 16)
Average finishing weight of chronic calves	--	0 (0, 1222)	1086 (1015, 1159)
Average daily gain (pounds per day) for chronic calves	--	0 (0, 3.97)	3.34 (3.10, 3.57)
Average days on feed for chronic calves	--	0 (0, 237)	163 (139, 190)
